# Composition and Antioxidant Activity of the Anthocyanins of the Fruit of *Berberis heteropoda* Schrenk

**DOI:** 10.3390/molecules191119078

**Published:** 2014-11-19

**Authors:** Li-Li Sun, Wan Gao, Meng-Meng Zhang, Cheng Li, Ai-Guo Wang, Ya-Lun Su, Teng-Fei Ji

**Affiliations:** State Key Laboratory of Bioactive Substance and Function of Natural Medicines, Institute of Materia Medica, Chinese Academy of Medical Sciences and Peking Union Medical College, Beijing 100050, China; E-Mails: sunlili@imm.ac.cn (L.-L.S.); gaowan@imm.ac.cn (W.G.); chenxiangmengmeng@163.com (M.-M.Z.); lc6722821@126.com (C.L.); wangaiguo@imm.ac.cn (A.-G.W.); suyalun@imm.ac.cn (Y.-L.S.)

**Keywords:** *Berberis heteropoda* Schrenk, HPLC-DAD, HPLC-HR-ESI-MS, TAC, DPPH, FRAP, ABTS

## Abstract

In present study, the anthocyanin composition and content of the fruit of *B. heteropoda* Schrenk were determined for the first time. The total anthocyanins were extracted from the fruit of *B. heteropoda* Schrenk using 0.5% HCl in 80% methanol and were then purified using an AB-8 macroporous resin column. The purified anthocyanin extract (PAE) was evaluated by high-performance liquid chromatography with a diode array detector (HPLC-DAD) and HPLC-high resolution-electrospray ionization-mass spectrometry (HPLC-HR-ESI-MS) under the same experimental conditions. The results revealed the presence of seven different anthocyanins. The major anthocyanins purified by preparative HPLC were confirmed to be delphinidin-3-O-glucopyranoside (30.3%), cyanidin-3-O-glucopyranoside (33.5%), petunidin-3-Ο-glucopyranoside (10.5%), peonidin-3-O-glucopyranoside (8.5%) and malvidin-3-O-glucopyranoside (13.8%) using HPLC-HR-ESI-MS and NMR spectroscopy. The total anthocyanin content was 2036.6 ± 2.2 mg/100 g of the fresh weight of *B. heteropoda* Schrenk fruit. In terms of its total reducing capacity assay, DPPH radical-scavenging activity assay, ferric-reducing antioxidant power (FRAP) assay and ABTS radical cation-scavenging activity assay, the PAE also showed potent antioxidant activity. The results are valuable for illuminating anthocyanins composition of *B. heteropoda* Schrenk and for further utilising them as a promising anthocyanin pigment source. This research enriched the chemical information of *B. heteropoda* Schrenk.

## 1. Introduction

*Berberis heteropoda* Schrenk, a type of Berberidaceae deciduous shrub that is native to the Xinjiang Uygur Autonomous Region of China, is used in both medicine and food [1]. Its ripe fruit contains glucose, fructose, malic acid, carotene, pigments and other substances. It was reported that *B. heteropoda* Schrenk is a rich source of anthocyanin compounds and that its beneficial effects are remarkable [2]. Its ripe fruits had been used for treatment of dysentery, enteritis, pharyngitis, stomatitis, eczema and hypertension [3]. Moreover, the residents of Kazakh and Uighur have drunk tea made from it for a long time. They also made it into jams. Thus, the value of the *B. heteropoda* Schrenk fruit has great potential for development. To date, only limited literature about this fruit is available, and most of it has focused on extraction methods, optimization of technical conditions, determination of the total flavonoid/berberine content, stability analyses or antitumor effects. However, the major anthocyanin constituents and the antioxidant activity of the fruit of *B. heteropoda* Schrenk have not yet been systematically studied, and in terms of large-scale applications, the potential of plant is now basically untapped [2]. This situation has largely restricted the research and development of the fruit of *B. heteropoda* Schrenk.

Anthocyanins are the most common pigmented flavonoids and are widespread in the plant kingdom. These compounds are responsible for most of the brilliant colors (orange, red, pink, purple and blue) observed in most fruits, flowers, leaves and cereal grains [4]. The naturally occurring anthocyanins of plants are cyanidin, delphinidin, peonidin, petunidin, malvidin, and pelargonidin [4,5]. From a chemical point of view, the anthocyanin molecule is composed of a flavylium nucleus bearing one or more sugar residues. The most prevalent of these sugars, including d-glucose, d-galactose, l-rhamnose, d-xylose, and d-arabinose, are 3-glycosides or 3,5-di-glycosides [6,7]. In addition, these sugars may be esterified by aliphatic or aromatic organic acids, which can greatly help to stabilize the anthocyanin structure. However, anthocyanins are not stable and are prone to degradation. The stability of anthocyanins is affected by several factors, such as their chemical structure, their concentration, oxygen, temperature, pH, light, enzymes, metal ions and various storage conditions [8].

In addition to their natural colorants role, anthocyanins have attracted considerable global interest, mainly due to their health-promoting benefits, such as reducing the risk of coronary heart disease and preventing several chronic diseases, which are associated with their antioxidant properties. The likely mechanism postulated for their effects is that anthocyanins act as potent antioxidants by scavenging free radical species such as reactive oxygen species (ROS) or reactive nitrogen species (RNS), inhibiting certain enzymes or chelating trace metals involved in free-radical production and upregulating or protecting antioxidant defenses, breaking the free-radical chain reaction [9,10]. Because of their beneficial health effects as dietary antioxidant, dietitians have proposed that adding certain amounts of exogenous anthocyanins derived from natural sources to the daily diet will delay or prevent many degenerative diseases.

Identifying anthocyanins using HPLC-DAD is complicated by the fact that some anthocyanins show similar retention times and spectroscopic characteristics [11]. In the past several decades, a good number of technological advances in HPLC and the introduction of the ultra-performance liquid chromatography (UPLC) have achieved significant improvements in both speed and separation of anthocyanins. In addition, advancements in mass spectrometry (MS), such as tandem MS, high-resolution MS (HR-MS) and sequential collision MS (MSn) allow fast structural elucidation of anthocyanins that play a important role in food anthocyanin research [12]. Due to their “soft” ionization properties, leading to the production of intact molecular ions and the corresponding anthocyanidin fragments, ESI-MS techniques have been shown to be highly suitable for anthocyanin characterization. Now, HPLC assisted by MS (HPLC-MS) has been frequently used as an excellent tool for the simultaneous chemical separation and identification of anthocyanin compounds. To our knowledge, the anthocyanin composition of the fruit of *B. heteropoda* Schrenk has never been described. One of the objectives of this study was to identify and characterize the anthocyanins of the fruit of *B. heteropoda* Schrenk using HPLC-HR-ESI-MS/MS. Another objective was to evaluate the antioxidant activity of its extracts according to the total reducing capacity, the DPPH-radical scavenging activity, the ferric-reducing antioxidant power (FRAP) and ABTS radical-cation scavenging activity.

## 2. Results and Discussion

### 2.1. Total Anthocyanin Content (TAC)

The amount of total anthocyanin in the PAE that was determined using the pH differential method was 2036.6 ± 2.2 mg/100 g of fresh weight of *B. Heteropoda* Schrenk fruit, expressed as cyanidin-3-O-glucoside equivalents and calculated as the mean value of three measurements and the standard deviation. This value is considerably higher than that of other anthocyanin-rich fruits and vegetables. For instance, the anthocyanin content of *Berberis boliviano* Lecher fruit is 1,500 mg/100 g of fresh fruit weight [13], that of black chokeberries is 560 mg/100 g of fresh fruit weight [13], that of wild *Lycium* *ruthenicum* Murr. from Dulan is 520 mg/100 g of fresh fruit weight, that of wild *Lycium* *ruthenicum* Murr. from Gomud is 470 mg/100 g of fresh fruit weight, and that of wild *Lycium* *ruthenicum* Murr. from Delingha is 475 mg/100 g of fresh fruit weight [4]. The anthocyanin content of each of the above fruits was lower than that of the fresh fruit of *B. heteropoda* Schrenk.

### 2.2. HPLC Analysis

**Figure 1:**
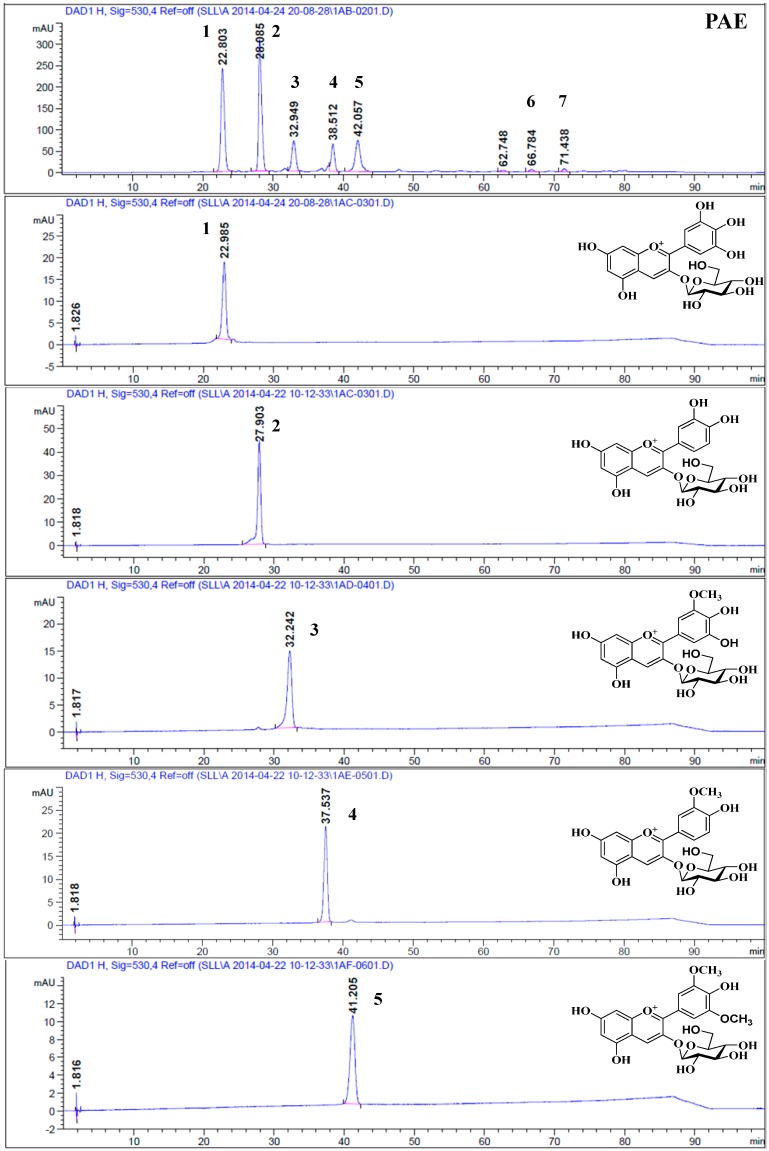
HPLC chromatogram of the five major anthocyanins of the fruit of *B. heteropoda* Schrenk that were detected at 530 nm. The peaks were numbered in order of their elution.

The HPLC-DAD chromatogram of the PAE of *B. heteropoda* Schrenk fruit that was obtained at 530 nm is displayed in Figure 1. The method utilized provided repeatable and satisfactory separation of the components of the PAE. Although several other components of the PAE were detected in the present study, five major anthocyanins were discovered as shown in the HPLC chromatogram. The major anthocyanins represented approximately 97% of the total peak area, and the relative amounts of anthocyanins 1–5 were approximately 30.3%, 33.5%, 10.5%, 8.5% and 13.8%, respectively. However, two minor peaks (Compounds **6** and **7**) were also detected which were identified as peonidin and malvidin by HPLC and HPLC-HR-ESI-MS/MS (Table 1).

### 2.3. Structural Elucidation

**Figure 2:**
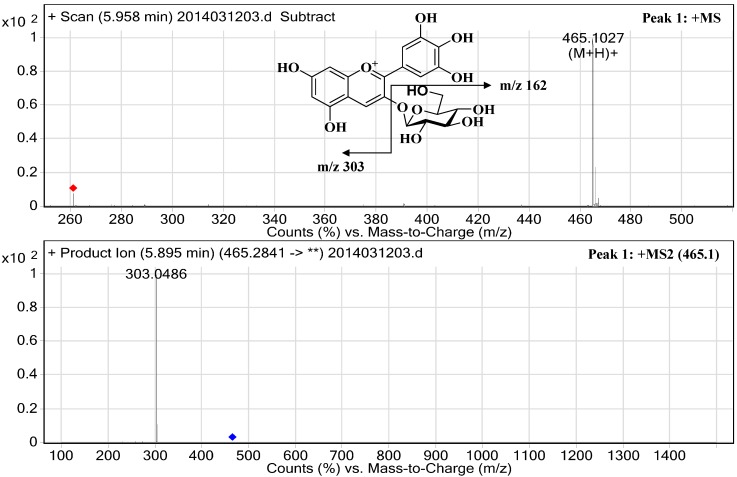

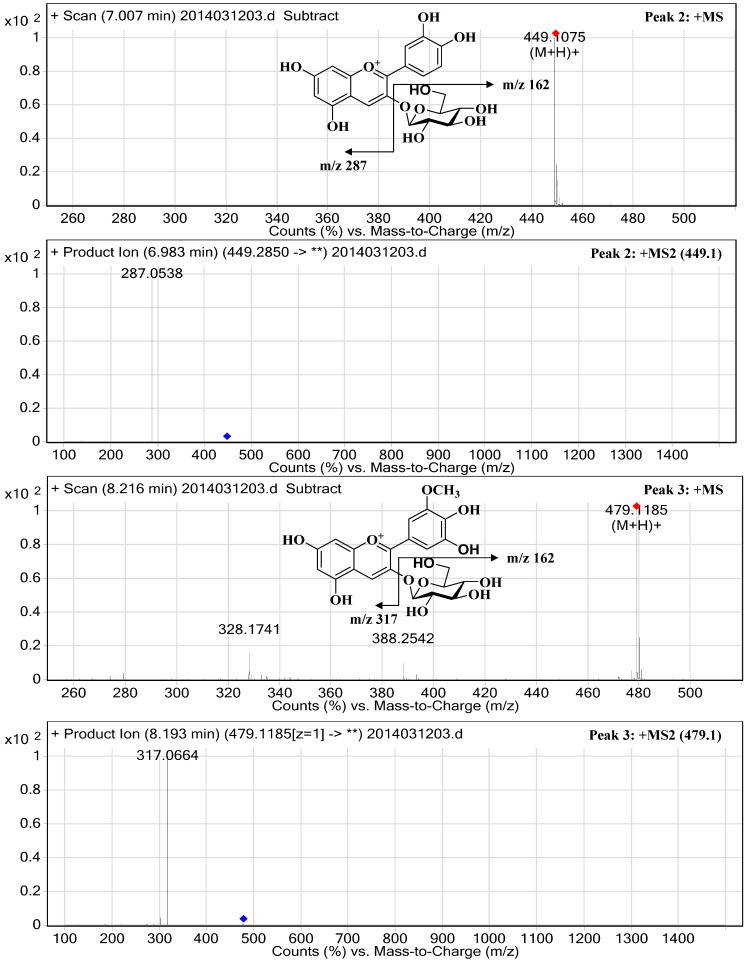

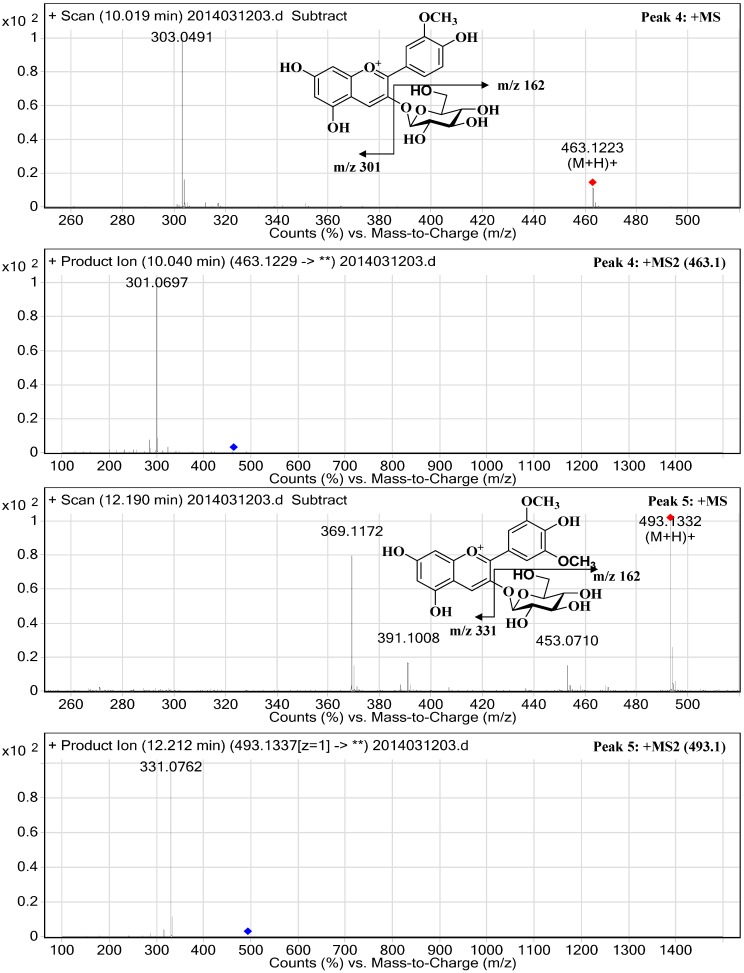
High-resolution electrospray mass spectrum of identified anthocyanins. Peak 1: delphinidin-3-O-glucopyranoside; Peak 2: cyanidin-3-O-glucopyranoside; Peak 3: petunidin-3-O-glucopyranoside; Peak 4: peonidin-3-O-glucopyranoside; and Peak 5: malvidin-3-O-glucopyranoside.

Five major anthocyanins were successfully isolated for the first time from the fruit of *B. heteropoda* Schrenk using preparative HPLC techniques. Compounds **1**–**5** were identified by HPLC-HR-ESI-MS/MS and NMR spectroscopic analysis. Figure 1 and Figure 2 shows the structures of these five anthocyanins.

Compound **1** was obtained as an amorphous red power. Its molecular formula of C_21_H_21_O_12_^+^ (calculated *m*/*z* 465.1034), was established based on the molecular ion at *m*/*z* 465.1027 [M+H]^+^ observed in the positive-ion HPLC-HR-ESI-MS/MS spectrum_._ A major fragmentation that occurred at *m*/*z* 303.0486 [M+H−162]^+^ was in accordance with the presence of a delphinidin aglycone and the loss of a hexose moiety. The ^1^H-NMR spectrum revealed a symmetrical aromatic proton signal at *δ* 7.71 (H-2'/H-6', s), two *meta*-coupled doublet protons on the A-ring at *δ* 6.63 (H-6, *J* = 1.5 Hz) and *δ* 6.82 (H-8, *J* = 1.5 Hz) and a singlet proton at *δ* 8.91 (H-4), suggesting the presence of a delphinidin nucleus. The proton signals observed at *δ* 5.30–3.50 (H-1-Glu-H-6-Glu) indicated the presence of a glucopyranose moiety. The anomeric proton signal at *δ* 5.30 (H-1-Glu, d, *J* = 7.5 Hz) in addition to the C-3 carbon signal at *δ* 144.9 ppm indicated that a sugar moiety with a *β*-configuration was attached to the C-3 position. From the above spectral studies, the structure of compound **1** was determined to be that of delphinidin-3-*O*-*β*-glucopyranoside, which corresponded with data in the literature [14].

Compound **2** was isolated as an amorphous red power. Its molecular formula was established as C_21_H_21_O_11_^+^ (calculated *m*/*z* 449.1082), with twelve degrees of unsaturation, based on its positive-ion HPLC-HR-ESI-MS/MS analyses. A molecular ion at *m*/*z* 449.1075 [M+H]^+^ and a major fragmentation occurring at *m/z* 287.0538 [M+H−162]^+^ corresponded to those of a cyanidin aglycone and a hexose unit. Similarly, the observation of a set of ABX-type aromatic proton signals at *δ* 7.02 (H-5', d, *J* = 8.5 Hz), *δ* 8.05(H-2', d, *J* = 2.0 Hz) and *δ* 8.23 (H-6', dd, *J* = 9.0, 2.5 Hz), two *meta*-coupled doublet protons on the A-ring at *δ* 6.65 (H-6, *J* = 1.5 Hz) and *δ* 6.87 (H-8, *J* = 1.0 Hz) and a singlet proton at *δ* 9.00 (H-4) in the ^1^H-NMR spectrum also implied the presence of a cyanidin nucleus. From the appearance of the anomeric proton signal at *δ* 5.28 (H-1-Glu, d, *J* = 7.5 Hz) and the C-3 carbon signal at *δ* 145.6 ppm indicated that a *β*-glucopyranose moiety was attached to C-3 position. Thus, compound **2** was elucidated as cyanidin-3-*O*-*β*-glucopyranoside by comparison of the spectral data with the literature [15].

Compound **3**, which also formed an amorphous red power, showed a molecular ion at *m/z* 479.1185 [M+H]^+^ and a major fragment ion at *m*/*z* 317.0664 [M+H−162]^+^ in the positive-ion HPLC-HR-ESI-MS/MS spectrum, which was in agreement with the molecular formula of C_22_H_23_O_12_^+^ (calculated *m/z* 479.1192), with twelve degrees of unsaturation. These data indicated the presence of a petunidin aglycon and a hexose moiety. Comparison of the ^1^H- and ^13^C-NMR spectra of compound **3** with those of compound **1** demonstrated that the structure of compound **3** was very similar to that of compound **1**, except for the presence of a methyl group [*δ*_H_ 3.98 (3H, s); *δ*_C_ 57.3]. Compound **3** was thus identified as petunidin-3-*O*-*β*-glucopyranoside by comparing these spectral data with reference [16,17].

Compound **4** was also presented as an amorphous red power. The positive-ion HPLC-HR-ESI-MS/MS spectrum showed its molecular ion at *m/z* 463.1223 [M+H]^+^ and a major fragment at *m/z* 301.0697 [M+H−162]^+^, corresponding to the molecular formula of C_22_H_23_O_11_^+^ (calculated *m/z* 463.1229), with twelve degrees of unsaturation. These data corresponded to those of a peonidin aglycone and a hexose moiety. Further support for this structure was obtained by comparing the ^1^H and ^13^C-NMR spectra of compound **4** with those of compound **2**. The spectral features of compound **4** were almost identical with those of compound **2**, except for the presence of a methyl group [*δ*_H_ 3.99 (3H, s); *δ*_C_ 56.7]. Based on the above-described observations, compound **4** was assigned as peonidin-3-*O*-*β*-glucopyranoside, which in accordance with the relevant published data [18].

Compound **5** was also obtained as an amorphous red power and had the molecular formula of C_23_H_25_O_12_^+^ (calculated *m/z* 493.1339), with twelve degrees of unsaturation, as determined using positive-ion HPLC-HR-ESI-MS/MS. A molecular ion at *m/z* 493.1332 [M+H]^+^ and a major fragment at *m/z* 331.0762 [M+H−162]^+^ were consistent with a structure consisting of a malvidin aglycone and a hexose moiety. By comparing their ^1^H and ^13^C-NMR spectra, the structure of compound **5** was found to differ from that of compound **1** mainly in the substitution pattern in ring B. The existence of a methyl group at *δ*_H_ 3.99 (6H, s, OCH_3_-3'/OCH_3_-5') in addition to a carbon signal at *δ*_C_ 56.7 ppm and a symmetrical aromatic proton signal at *δ* 7.97 (H-2'/H-6', s) revealed two methoxyl groups, one located at C-3 and the other at C-5. Based on the above-described evidence, compound **5** was identified as malvidin-3-*O*-*β*-glucopyranoside by comparison with previous data [19].

The detailed data obtained using HPLC-HR-ESI-MS/MS are presented in Table 1 and Figure 2, and the ^1^H- and ^13^C-NMR spectroscopic data are shown in Table 2. This is the first time that all of the major anthocyanins in *B. heteropoda* Schrenk fruits were systematically isolated and characterized.

**Table 1:** Chromatographic and spectroscopic characteristics of the major anthocyanins in the fruits of *B. heteropoda* Schrenk, determined using HPLC-DAD and HPLC-HR-ESI-MS/MS.

Peak ^a^	t_R_ (min) HPLC	(%) HPLC	λ_max_ (nm)	t_R_ (min) HPLC-HR-ESI-MS/MS	M^+^ (*m/z*)	Fragment Ions (*m/z*)	Compound	Contents ^b^ (mg/100 g Fresh Fruits)
1	22.38	30.34	524/278	5.96	465.1027	303.0486	Delphinidin-3-O-glucopyranoside	617.84 ± 0.98
2	27.54	33.47	516/280	7.01	449.1075	287.0538	Cyanidin-3-O-glucopyranoside	681.58 ± 1.13
3	34.42	10.55	527/278	8.22	479.1185	317.0664	Petunidin-3-O-glucopyranoside	212.59 ± 1.79
4	37.88	8.53	520/280	10.02	463.1223	301.0697	Peonidin-3-O-glucopyranoside	173.65 ± 0.66
5	41.48	13.77	528/278	12.20	493.1332	331.0762	Malvidin-3-O-glucopyranoside	279.83 ± 0.60
6	78.83	0.16	520/280	26.12	301.0704	-	Peonidin	3.29 ± 0.05
7	79.52	0.22	530/278	27.38	331.0803	-	Malvidin	4.49 ± 0.06

Notes:^ a^ Numbered according to the order of elution; ^b^ mean value ± SD (*n* = 3).

**Table 2:** ^1^H-NMR and ^13^C-NMR spectroscopic data for compounds **1**–**5** in CD_3_OD/TFA-d (7:1, v/v) (*δ* in ppm. *J* in Hz).

Position	1		2		3		4		5	
	*δ*_H _*^a^*	*δ*_C _*^c^*	*δ*_H _*^a^*	*δ*_C _*^d^*	*δ*_H _*^b^*	*δ*_C _*^d^*	*δ*_H _*^a^*	*δ*_C _*^c^*	*δ*_H _*^a^*	*δ*_C _*^d^*
2		164.1		164.4		164.0		164.1		163.0
3		144.9		145.6		145.1		145.5		145.3
4	8.91 (s)	136.4	9.00 (s)	137.1	8.98 (s)	136.9	9.02 (s)	137.4	9.02 (s)	137.3
5		159.6		159.6		159.0		158.8		158.9
6	6.63 (d, 1.5)	103.9	6.65 (d, 1.5)	103.8	6.67 (d, 1.2)	103.6	6.66 (d, 1.5)	103.5	6.67 (d, 0.5)	103.1
7		170.8		170.7		170.7		170.9		170.9
8	6.82 (d, 1.5)	95.4	6.87 (d, 1.0)	95.2	6.88 (d, 0.6)	95.2	6.89 (d, 1.5)	95.3	6.93 (d, 0.5)	95.4
9		157.9		157.8		157.8		157.9		157.9
10		112.9		113.8		113.6		113.6		112.9
1’		120.3		121.3		120.1		121.1		119.9
2'	7.71 (s)	113.6	8.05 (d, 2.0)	118.6	7.90 (d, 1.8)	109.2	8.19 (d, 1.5)	115.2	7.97 (s)	110.6
3’		147.7		147.4		149.9		149.6		149.8
4’		146.1		155.7		145.7		156.5		164.4
5'		147.7	7.02 (d, 8.5)	117.3		147.4	7.05 (d, 9.0)	117.6		149.8
6'	7.71 (s)	113.6	8.23 (dd, 9.0, 2.5)	128.1	7.81 (d, 1.8)	112.9	8.23 (dd, 8.5, 2.0)	128.9	7.97 (s)	110.6
3'-O Me					3.98 (s)	57.3	3.99 (s)	56.7	3.99 (s)	56.7
5'-O Me									3.99 (s)	56.7
1''	5.30 (d, 7.5)	103.7	5.28 (d, 7.5)	104.1	5.32 (d, 7.8)	103.8	5.29 (overlap)	103.9	5.33 (overlap)	103.9
2''	3.77 (m)	75.0	3.73 (m)	74.8	3.70 (m)	74.9	3.67 (m)	74.9	3.71 (m)	74.9
3''	3.61 (m)	78.3	3.56 (m)	78.1	3.57 (m)	78.2	3.60 (m)	78.2	3.60 (m)	78.1
4''	3.50 (m)	71.3	3.46 (m)	71.1	3.47 (m)	71.1	3.47 (m)	71.2	3.47 (m)	71.3
5''	3.61 (m)	79.0	3.56 (m)	78.7	3.57 (m)	78.8	3.60 (m)	78.8	3.60 (m)	77.8
6''a	3.94 (dd, 12.0, 1.5)	62.6	3.92 (dd, 12.0, 2.0)	62.4	3.94 (dd, 9.0, 3.0)	62.3	3.93 (dd, 10.0, 2.0)	62.4	3.92 (dd, 9.5, 1.5)	62.3
6''b	3.77 (dd, 12.5, 5.0)		3.73 (dd, 12.0, 4.5)		3.74 (dd, 12.0, 6.0)		3.72 (dd, 12.5, 6.0)		3.77 (dd,12.0, 6.0)	

Notes: *^a^* The ^1^H-NMR data were obtained at 500 MHz ; *^b^* The ^1^H-NMR data were obtained at 600 MHz; *^c^* The ^13^C-NMR data were obtained at 125 MHz; *^d^* The ^13^C-NMR data were obtained at 150 MHz.

### 2.4. Antioxidant Activity

#### 2.4.1. Total Reducing Capacity of the PAE

**Figure 3:**
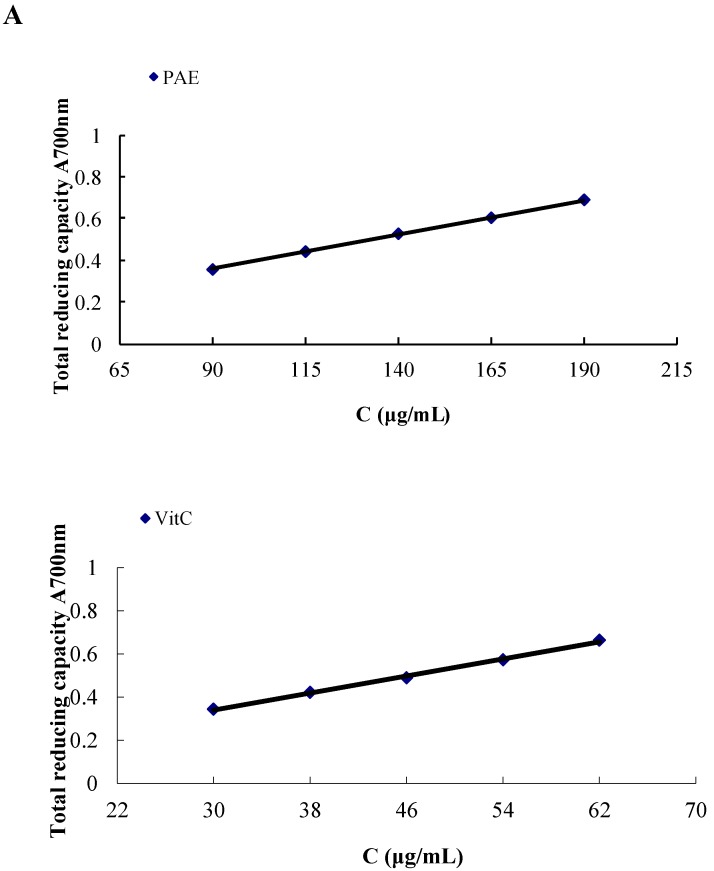

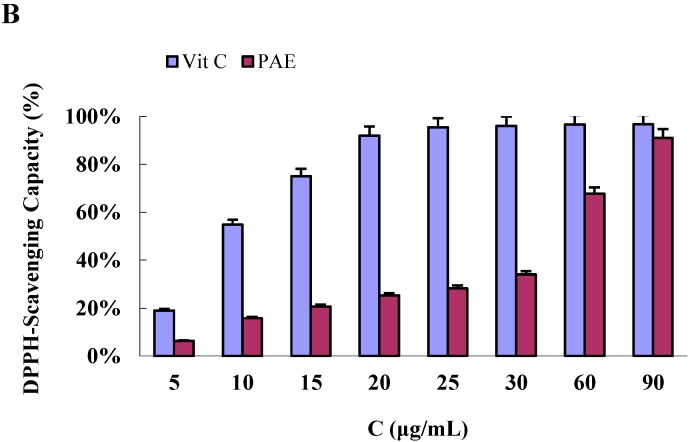
Antioxidant activities of the PAE of *B. heteropoda* Schrenk fruit determined using the total reducing capacity assay (**A**) and the DPPH free radical-scavenging assay (**B**). The data represented by different-colored bars are significantly different (*p* < 0.05).

The total reducing power of the anthocyanins can be used as an index of their antioxidative electron-donating activity. The total reducing capacity of the PAE and the ascorbic-acid control are shown in Table 3 and Figure 3A, respectively. The results showed that the reducing power of the PAE was approximately one-third that of ascorbic acid. The IC_50_ value, which was the concentration that raised the absorbance at 700 nm to 0.5, of the reducing power of the PAE was 139.65 μg/mL. Furthermore, both PAE and ascorbic acid exhibited a dose-dependent reducing power. Good linearity was obtained using the PAE at concentrations of 90 to 190 μg/mL and ascorbic acid at concentrations of 30 to 62 μg/mL. The calibration curve of the PAE and ascorbic acid were Y (absorbance) = 0.0033X (concentration) + 0.0615 (r = 0.999), Y (absorbance) = 0.0099X (concentration) + 0.0434 (r = 0.995), respectively.

**Table 3:** Antioxidant activities of PAE and ascorbic acid in terms of the total reducing capacity assay, DPPH radical scavenging activity assay, ferric-reducing antioxidant power (FRAP) assay and ABTS radical cation scavenging activity assay.

Samples	A_700 nm_ = 0.5/Total Reducing Power (μg/mL) ^a^	IC_50_/DPPH (μg/mL) ^b^
PAE	139.65 ± 0.01	47.16 ± 0.35
Ascorbic acid	46.12 ± 0.04	9.30 ± 0.21
Samples	FRAP value (mmol/g)	TEAC/ABTS
PAE	4.32 ± 0.03	2.250 ± 0.17
Trolox	7.007 ± 0.14	-

Notes: All the trials were performed in triplicate and all the data represent the means ± standard deviation (*n* = 3). Data in the same column with different letters are significantly different (*p* < 0.05); ^a^ The antioxidant activity was evaluated as the concentration of the test sample required to raise the absorbance at 700 nm to 0.5; ^b^ The antioxidant activity was calculated as the concentration of the test sample required to decrease the absorbance at 517 nm by 50%.

#### 2.4.2. DPPH Radical-Scavenging Activity of the PAE

The effect of an antioxidant on DPPH radical scavenging is generally ascribed to its hydrogen-donating ability. The decrease in absorbance at 517 nm reflects the DPPH-radical reduction potential of samples. In the present study, the concentration of the samples required to scavenge 50% of the DPPH radicals (IC_50_) was used as an indicator for comparing their antioxidant activities. The results are shown in Table 3 and Figure 3B. As shown in Figure 3B, the DPPH radical-scavenging activity of the PAE and ascorbic acid increased as the concentrations increased. At a concentration of 90 μg/mL, the PAE scavenged almost all of the DPPH radicals that were present (>95%). The IC_50_ value for the PAE was 47.16 μg/mL, however, the IC_50_ value for the control, ascorbic acid, was 9.30 μg/mL. In terms of the IC_50_ value, DPPH radical-scavenging activity of the PAE was approximately one-fifth that of ascorbic acid.

#### 2.4.3. Ferric-Reducing Antioxidant Power (FRAP) of the PAE

A simple and reliable assay was adopted to evaluate the reducing capacity of antioxidants in the present study. This assay is based on the reaction of an antioxidant with the TPTZ-Fe(III) complex to generate TPTZ-Fe(II). The absorbance of TPTZ-Fe(II) at 593 nm was measured to evaluate the reducing power of the tested sample. The corresponding FeSO_4_ value provides quantification of the antioxidant activity of the extract. A higher FeSO_4_ value demonstrates a higher ferric-reducing capacity. In this assay, the PAE exhibited a high antioxidant activity, with a FeSO_4_ value of 4.32 mmol/g, whereas the FeSO_4_ value of the Trolox control was determined to be 7.007 mmol/g (Table 3). As far as the FeSO_4_ value be concerned, Ferric-reducing capacity of PAE was approximately three-fifths that of Trolox.

#### 2.4.4. ABTS^+^ Radical-Scavenging Activity of the PAE

The ABTS^+^ radical-scavenging assay is considered an excellent tool for investigating the antioxidant activity of hydrogen-donating antioxidants and chain-breaking antioxidants [20]. This assay is based on the inhibitory effect of the antioxidant on the absorbance of ABTS^+^, which reflects the antioxidant capacity of the tested sample. The TEAC values were calculated using standard curves, with a higher TEAC value indicating a higher antioxidant activity. The PAE exhibited a good antioxidative activity in this test, with a TEAC value of 2.25 mmol/g (Table 3), which was comparable to that of the extract of pigeon-pea leaves (1.095 mmol/g) [21]. Therefore, *B. heteropoda* Schrenk fruit is an excellent candidate antioxidant.

## 3. Experimental Section

### 3.1. Plant Material

Fresh ripe fruit (3 kg) of *B. heteropoda* Schrenk was manually harvested in Daxigou (Latitude 44°26′ N, Longitude 80°46′ E, Altitude 1000 m), Huocheng County, Yili Kazakh Autonomous Prefecture, Xinjiang Uygur Autonomous Region of China during September 2012. The fruit was maintained at −18 °C from immediately after collection until they were used. Samples were also preserved in a refrigerator (−18 °C) for later analysis in the laboratory.

### 3.2. Reagents and Apparatus

2,2-Diphenyl-1-picrylhydrazyl (DPPH), potassium ferricyanide and ascorbic acid (vitamin C) were purchased from the Sigma-Aldrich Chemical Co. (Shanghai, China); The FRAP and ABTS assay kits were obtained from the Beyotime Institute of Biotechnology (Haimen, Jiangsu Province, China). The methanol and acetonitrile for the HPLC analysis were of chromatographic grade and were purchased from J.T. Baker (Phillipsburg, NJ, USA). All other reagents and solvents were of analytical grade.

The ultrasonicator was purchased from Tian Pong Electricity New Technology Co. Ltd. (Beijing, China) The filter papers were produced by Hangzhou Special Paper Industry (Hangzhou, Zhejiang Province, China). The 0.45-μm reinforced nylon membrane filter was purchased from ANPEL (Shanghai, China). The freeze dryer used was an EYELA FDU-1100 model (Tokyo, Japan). The L5S spectrophotometer was purchased from the Jingke Industrial Co. Ltd. (Shanghai, China). The multimode reader was a PerkinElmer EnSpire model (Waltham, MA, USA).

### 3.3. Extraction and Primary Purification of the Anthocyanins

The fresh fruit of *B. heteropoda* Schrenk (100 g) were crushed using a pulverizer, and then ultrasonically extracted for 30 min using 1 L of 0.5% HCL (v/v) in 80% methanol in the dark at room temperature. This extraction process was repeated three times to ensure exhaustive extraction. The supernatants were combined and were filtered through filter paper to remove the fruit residues, proteins and the polysaccharide-containing sediment. The filtrate was concentrated using a rotary evaporator at less than 35 °C, and then was lyophilized to produce the crude anthocyanin extract (22.666 g).

The crude anthocyanin extract was purified using an AB-8 macroporous resin column (2.5 cm × 45 cm) to remove sugars, acids and other water-soluble substances. The successive eluting agents were 0.5% aqueous HCl (1 L), 0.5% HCl in 95% ethanol (1 L), 95% ethanol (1 L). The eluent obtained using 95% ethanol (0.5% HCl) was evaporated under reduced pressure at less than 35 °C and was freeze-dried to yield the PAE (10.956 g).

### 3.4. Isolation and Identification of the Main Anthocyanins in the PAE

#### 3.4.1. Isolation

The PAE (5.0 g) was further fractionated using a medium-pressure chromatographic column (4.9 × 46 cm) using a gradient elution of 0%, 20%, 25%, 30%, 35%, 40%, 50%, 70% CH_3_OH/H_2_O (containing 0.1% HCl; 2 L of each eluent), resulting in 37 fractions. The 37 fractions were concentrated separately under reduced pressure at less than 35 °C and then were analyzed using HPLC. Fractions 12 (121 mg), 15 (209 mg) and 21 (201 mg) were further refined by preparative HPLC (Shimadzu LC-6AD, Kyoto, Japan) using a YMC-Pack ODS column (20 × 250 mm, 10 μM, YMC Co. Ltd., Kyoto, Japan) at room temperature with a flow rate of 4.0 mL/min and detection at 530 nm. The eluting solvents were CH_3_CN/H_2_O/TFA at a ratio of 12:87.9:0.1, 11:88.9:0.1, 13:86.9:0.1, which yielded compounds **1** (21.0 mg), **2** (21.0 mg) and **3** (3.4 mg), **4** (3.0 mg) and **5** (5.0 mg).

#### 3.4.2. NMR Identification

The structures of the isolated anthocyanins were elucidated by spectroscopic analysis. ^1^H (500 MHz) and ^13^C (125 MHz) NMR spectra were obtained using an Inova 500 spectrometer (Agilent Technologies, Inc., Santa Clara, CA, USA) in CD_3_OD/CF_3_COOD (7:1, v/v) containing TMS as an internal standard. The chemical shift values were expressed in *δ* (ppm) and the coupling constant (*J*) values were presented in Hertz.

### 3.5. Total Anthocyanin Content

The TAC was determined using the pH differential method [22,23]. The absorbance at 530 nm and 700 nm was measured in pH 1.0 buffer (potassium chloride-hydrochloric acid) and pH 4.5 buffer (sodium acetate-acetic acid). The results were expressed as mg of cyanidin-3-glycoside equivalents/100 g of fresh fruit weight. The TAC was calculated using the following equation:

*A* = [(*A*_530_ − *A*_700_) _pH 1.0_ − (*A*_530_ − *A*_700_) _pH 4.5_] (1)

(2) $$\text{TAC (mg/100 g)}=A\times \text{MW}\times \text{DF}\times \frac{1}{\varepsilon \times L}\times \frac{V}{M}\times 100$$

where MW is the molecular weight of cyanidin-3-glucoside (449.2 g/mol), DF is the dilution factor, ε is the molar extinction coefficient of cyanidin-3-glucoside (26,900 L·cm^−1^·mol^−1^), *L* is the cell-path length (1 cm), *V* is the extract volume (mL) and *M* is the fresh fruit weight (g). The data were expressed as the mean values ± SD (*n* = 3).

### 3.6. HPLC-DAD Analysis

The anthocyanins were separated using an analytical HPLC system (Agilent 1290) equipped with a G4220A 1290 Bin Pump, a G4226A 1290 Sampler, a G1316C 1290 TCC and a G4212A 1290 DAD. The analytical column used was an Agilent ZORBAX Eclipse XDB C18 column (4.6 × 150 mm, 5 μm, Agilent). The PAE (10 mg/mL) was filtered through a 0.45-μm reinforced nylon membrane filter before injection. An aliquot of 5 μL of solution was injected. The anthocyanin chromatograms were obtained in the visible spectral region (530 nm), and the spectroscopic data from 200 to 600 nm were recorded throughout the entire run.

A gradient program was applied for analysis of the anthocyanins. This program followed a previously reported technique, with a slight modification [4]. The mobile phases were as follows: A, a 3% formic-acid aqueous solution and B, 15% methanol in acetonitrile. The applied gradient conditions were as follows: 0–40 min, linear gradient from 3% to 11.5% B; 40–50 min, 11.5% B; 50–60 min, linear gradient from 11.5% to 13.5% B; 60–70 min, linear gradient from 13.5% to 15.5% B; 70–85 min, linear gradient from 15.5% to 23% B; and 85–90 min, linear gradient from 23% to 3% B. The flow rate was 0.8 mL/min, and temperature was 35 °C. The peaks of the anthocyanins from *B. heteropoda* Schrenk fruit were numbered in order of their elution.

### 3.7. HPLC-DAD-HR-ESI-MS/MS

HPLC-DAD-HR-ESI-MS/MS was performed using an Agilent 6520 Accurate-Mass Q-TOF LC/MS. The chromatographic separation was conducted using an Agilent ZORBAX Eclipse XDB C18 column (4.6 × 150 mm, 5 μm, Agilent, Wilmintton, DE, USA). The separation conditions were the same as those used for HPLC/DAD analysis, described above. The concentration of the PAE was 1.0 mg/mL. The MS parameters were as follows: capillary voltage, 4000 V; gas (N_2_) temperature, 350 °C; flow rate, 8 L/min; and nebulizer pressure, 35 psi. The instrument was operated in the positive-ion mode with scanning from* m/z* 0 to 1500.

### 3.8. Antioxidant Activity

#### 3.8.1. Total Reducing Capacity Assay

The total reducing capacity of the PAE was evaluated as described by Cui* et al.*, with a slight modification [22]. For this analysis, the PAE was dissolved in distilled water for the preparation of solutions of various concentrations (90, 115, 140, 165, and 190 μg/mL). One milliliter of a sample solution was mixed with 2.5 mL of sodium phosphate buffer (0.2 M, pH 6.6) and 2.5 mL of 1% (w/v) potassium ferricyanide. The mixtures were incubated at 50 °C for 20 min in the dark and then was centrifuged at 1000 g for 10 min after adding 2.5 mL of 10% trichloroacetic acid. The supernatants (2.5 mL) were collected and mixed in a test tube with 2.5 mL of distilled water and 1.0 mL of 0.1% (w/v) ferric chloride. After a 10-min incubation at room temperature in the dark, the absorbance of the resulting solution at 700 nm was recorded using a spectrophotometer. An equivalent volume of distilled water, instead of the sample, was used as a control. The reducing power of ascorbic acid (30, 38, 46, 54, 62 μg/mL) was also determined for comparison. The increased absorbance of the reaction mixture at 700 nm indicated an increased reducing capacity. The concentration of the test sample that was required to raise the level of absorbance to 0.5 at 700 nm was calculated.

#### 3.8.2. DPPH Radical-Scavenging Activity Assay

A modified method was employed to evaluate the DPPH radical-scavenging activity [24]. Briefly, 2.0 mL of an ethanolic solution of PAE at various concentrations (5, 10, 15, 20, 25, 30, 60 and 90 μg/mL) was added to 2.0 mL of 0.2 mM DPPH that was dissolved in ethanol. The mixture was then shaken vigorously and was maintained for 30 min at room temperature in the dark. The absorbance of the mixed solution at 517 nm was determined. Ascorbic acid (vitamin C) was used as the reference compound. All of the samples were analyzed in triplicate. The scavenging activity of each sample was calculated according to the following formula:

Scavenging activity (%) = [1 − (A _DPPH sample_ − A _sample control_)/A_DPPH blank_] × 100 (3)

where A _DPPH sample_ = absorbance of 2 mL of the sample solution + 2 mL of DPPH solution; A _sample control_ = absorbance of 2 mL of the sample solution + 2 mL of ethanol; and A _DPPH blank_ = absorbance of 2 mL of ethanol + 2 mL of DPPH solution. The concentration of sample required to cause 50% inhibition (IC_50_ value) was determined.

#### 3.8.3. Ferric-Reducing Antioxidant Power (FRAP) Assay

The FRAP of the PAE was determined using a total antioxidant-capacity assay kit following the FRAP method (Beyotime Institute of Biotechnology, Haimen, China), using a previous report as a reference [25]. The stock solutions included a TPTZ (2,4,6-tripyridyl-s-triazine) solution, a TPTZ diluent, the detection buffer, 1.0 mL of a 100 mM FeSO_4_ solution and 0.1 mL of a 10 mM Trolox solution. A working solution was freshly prepared by mixing the TPTZ diluent, the TPTZ solution and the detection buffer in a 10:1:1 (v/v) ratio, respectively. The working solution was maintained at 37 °C before use. An aliquot of 5 μL of the PAE solution was allowed to react with 180 μL of the FRAP working solution for 3–5 min at 37 °C. Then, the absorbance of the mixture at 593 nm was measured using a multimode reader. Five microliters of distilled water, instead of a sample, was used for the blank. The standard curve was obtained using FeSO_4_ in the concentration range of 0.15–1.5 mM. The results were expressed as FeSO_4_ values, which were calculated using on the standard curve.

#### 3.8.4. ABTS^+^ Radical-Scavenging Capacity Assay

The ABTS^+^ radical-scavenging capacity was determined according to the instruction of the Beyotime Institute of Biotechnology and methods described previously [21]. This capacity is based on the ability of different substances to scavenge the ABTS^+^ radical in comparison with that of a standard (Trolox). The stock solutions included an ABTS solution and an oxidant solution. The green ABTS^+^ solution was prepared by reacting ABTS with an equal quantity of an oxidant in and allowing the mixture to stand for 12–16 h at room temperature in the dark before use. The resulting solution was diluted with 80% ethanol to obtain an absorbance of 0.70 ± 0.05 at 734 nm. The solution was prepared freshly for each assay. An aliquot of 10 μL of the PAE solutions was mixed with 200 μL of the diluted ABTS^+^ solution and maintained for 2–6 min at room temperature in the dark. Then, the absorbance of the mixture at 734 nm was recorded. A calibration curve was prepared using Trolox (a water-soluble analogue of vitamin E) in a range of concentrations from 0.15 to 1.5 mM.

To ensure a correct measurement, the test sample was diluted with water to the concentration at which the percentage of inhibition was 20%–80%. The result was calculated based on the Trolox standard curve and was expressed as the Trolox-equivalent antioxidant capacity (TEAC), which was defined as the mmol of Trolox for which the antioxidant activity was equivalent to the activity of 1 g of the sample.

### 3.9. Statistical Analysis

All of the assays of the antioxidant capacities (total reducing capacity, DPPH radical-scavenging activity, FRAP and ABTS radical-scavenging capacity) were performed in triplicate. The analytical data were expressed as the mean values from assays conducted in triplicates and the standard deviation (SD).

## 4. Conclusions

Our research has provided the chemical basis for and evidence of high levels of antioxidant activity of the fruit of *B. heteropoda* Schrenk. The high anthocyanins content indicated that the fruit of *B. heteropoda* Schrenk can be considered as an excellent source of natural colorants and a functional food that benefits human health However, we have only taken a glimpse into the antioxidant activity of the fruit of *B. heteropoda* Schrenk. Further investigations must be conducted to elucidate its other biological effects. In conclusion, the composition of the major anthocyanins and the antioxidant activities of the fruit of *B. heteropoda* Schrenk were systematically investigated for the first time. The results of this research are important for the further development of applications of the fruit of *B. heteropoda* Schrenk.

## Supplementary Materials

Supplementary materials can be accessed at: http://www.mdpi.com/1420-3049/19/11/19078/s1.
