# Supplementary material for: Composition and Antioxidant Activity of the Anthocyanins of the Fruit of Berberis heteropoda Schrenk
Source: Molecules. 2014 Nov 19;19(11):19078–96. doi: 10.3390/molecules191119078 (PMC6271762; doi:10.3390/molecules191119078)
Supplement: Supplementary file 1 [file molecules-19-19078-s001.pdf]

Supplementary Materials

NMR Data

Figure S1. ^{1}H-NMR Spectrum (500 MHz, CD3OD) of Compound **1**.

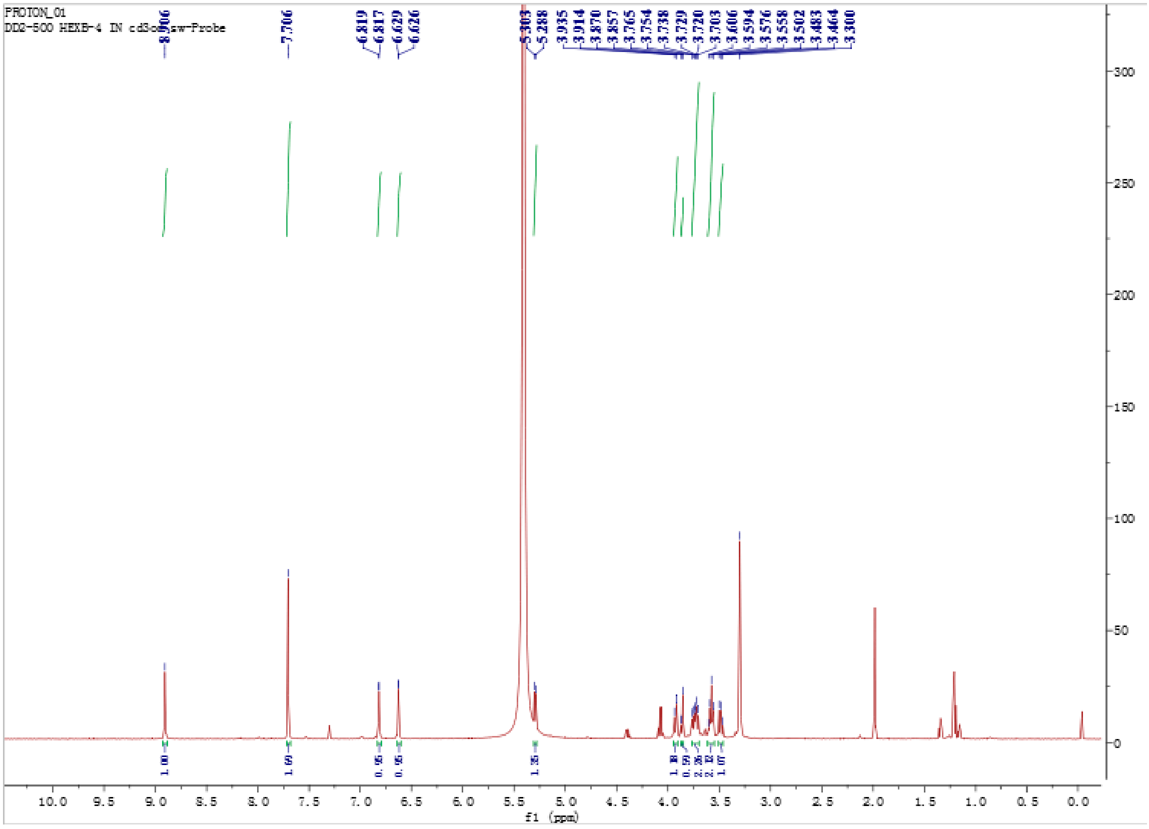

Figure S2. ^{13}C-NMR Spectrum (500 MHz, CD3OD) of Compound **1**.

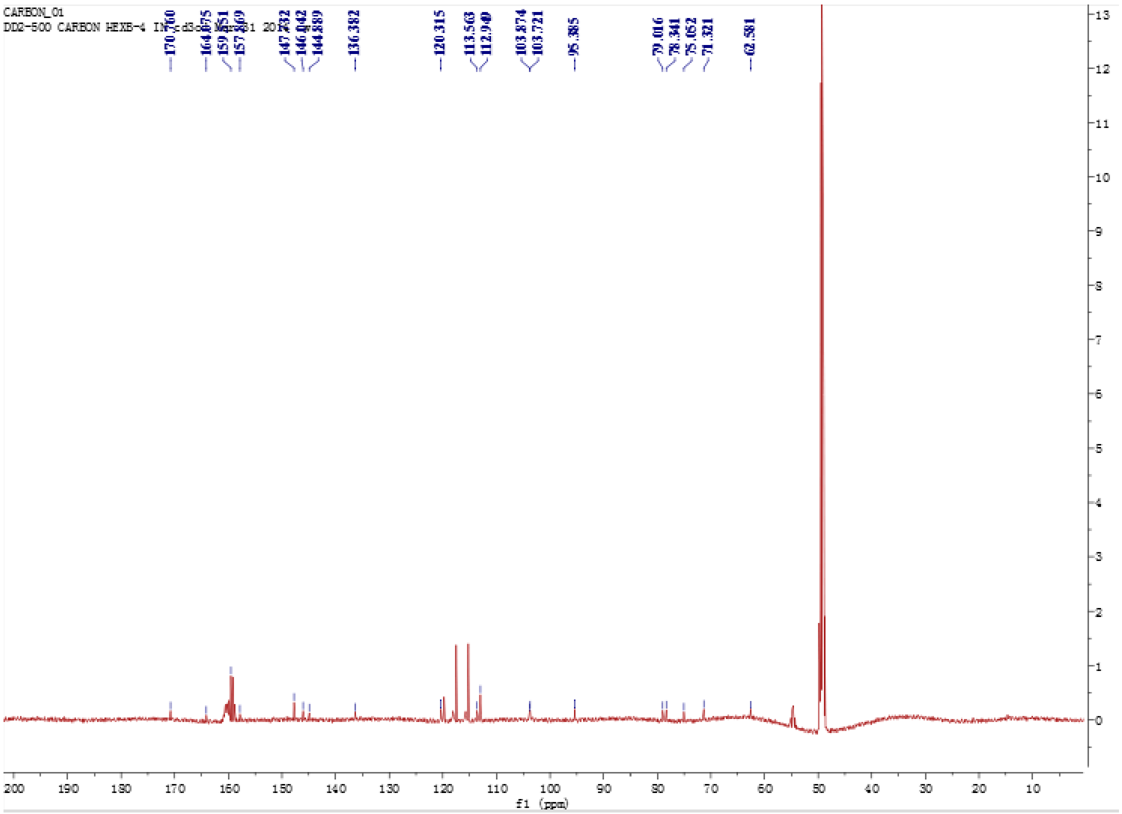

Figure S3. ^{1}H-NMR Spectrum (500 MHz, CD3OD) of Compound **2**.

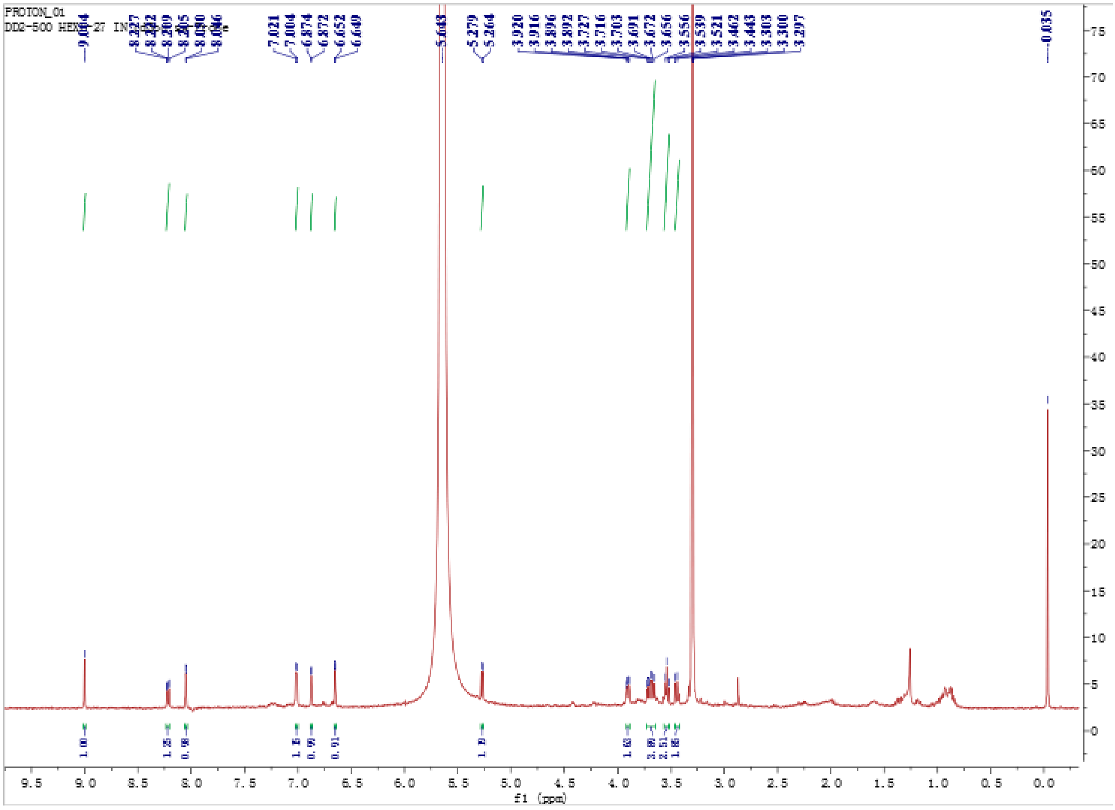

Figure S4. ^{13}C-NMR Spectrum (600 MHz, CD3OD) of Compound **2**.

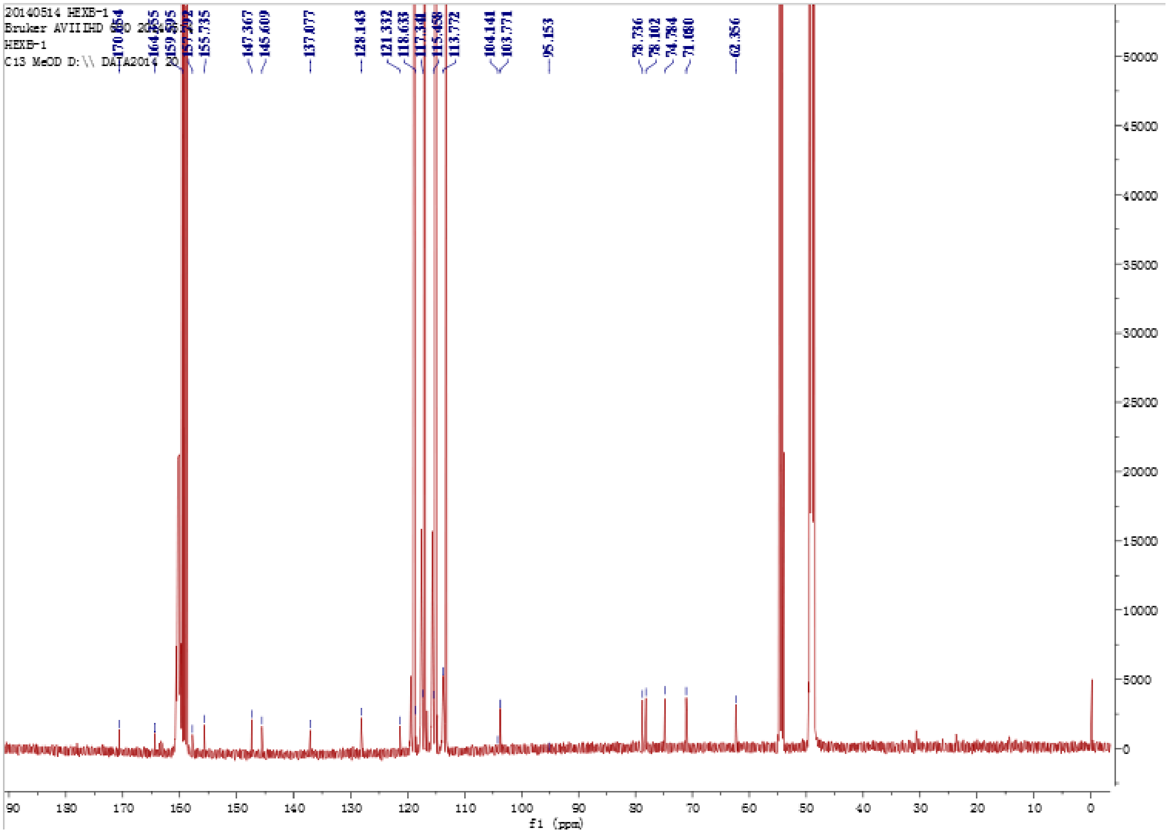

Figure S5. ^{1}H-NMR Spectrum (600 MHz, CD_3OD) of Compound **3**.

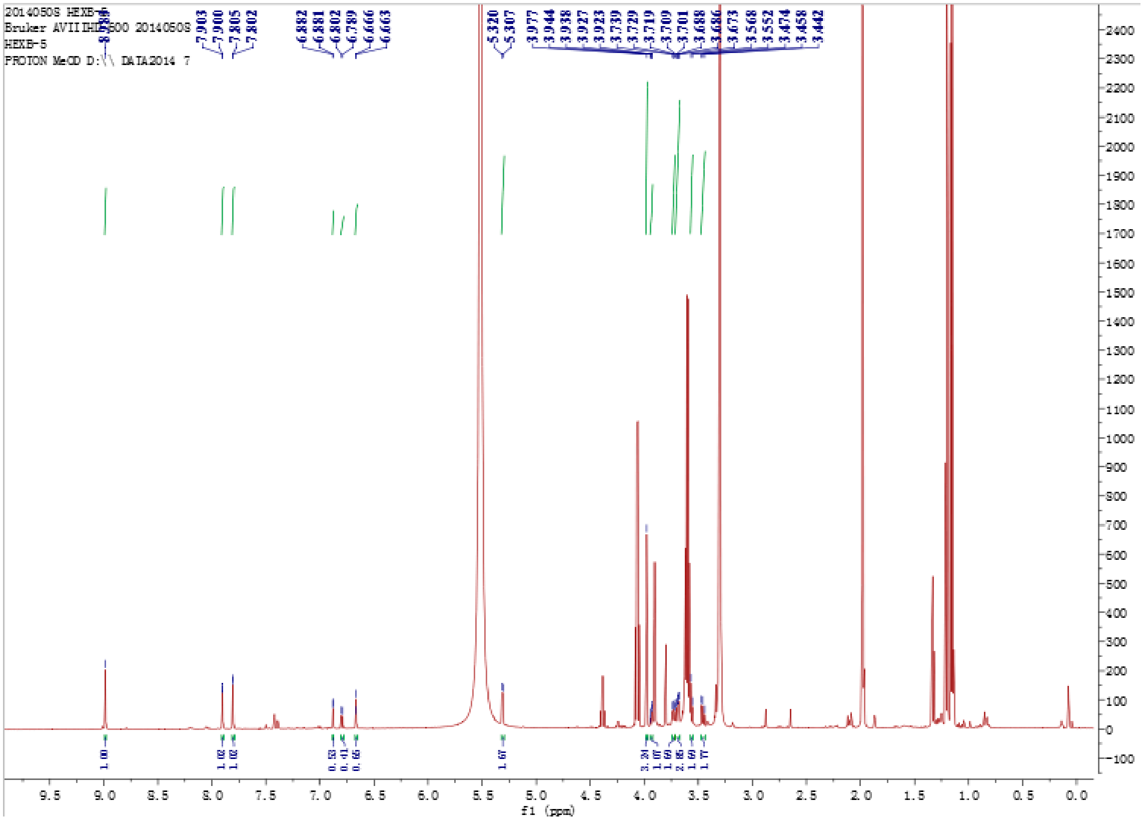

Figure S6. ^{13}C-NMR Spectrum (600 MHz, CD_3OD) of Compound **3**.

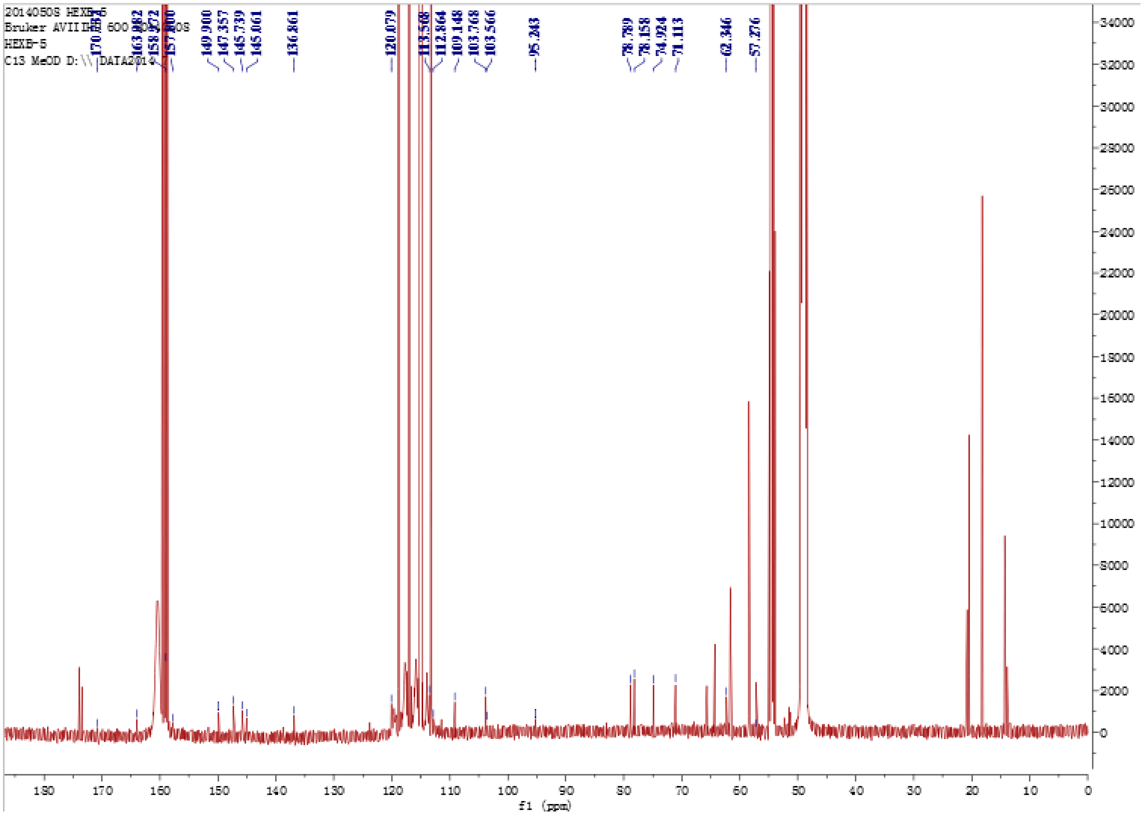

Figure S7. ^{1}H-NMR Spectrum (500 MHz, CD_3OD) of Compound **4**.

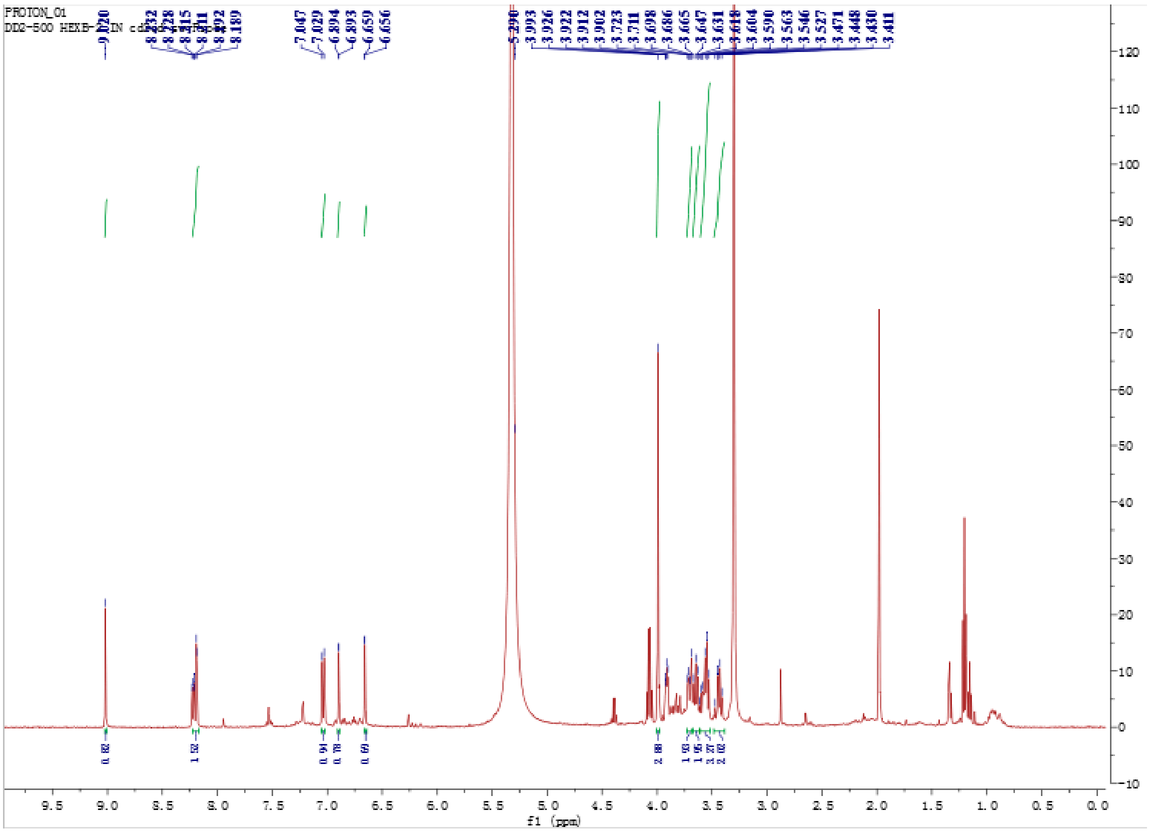

Figure S8. ^{13}C-NMR Spectrum (500 MHz, CD_3OD) of Compound **4**.

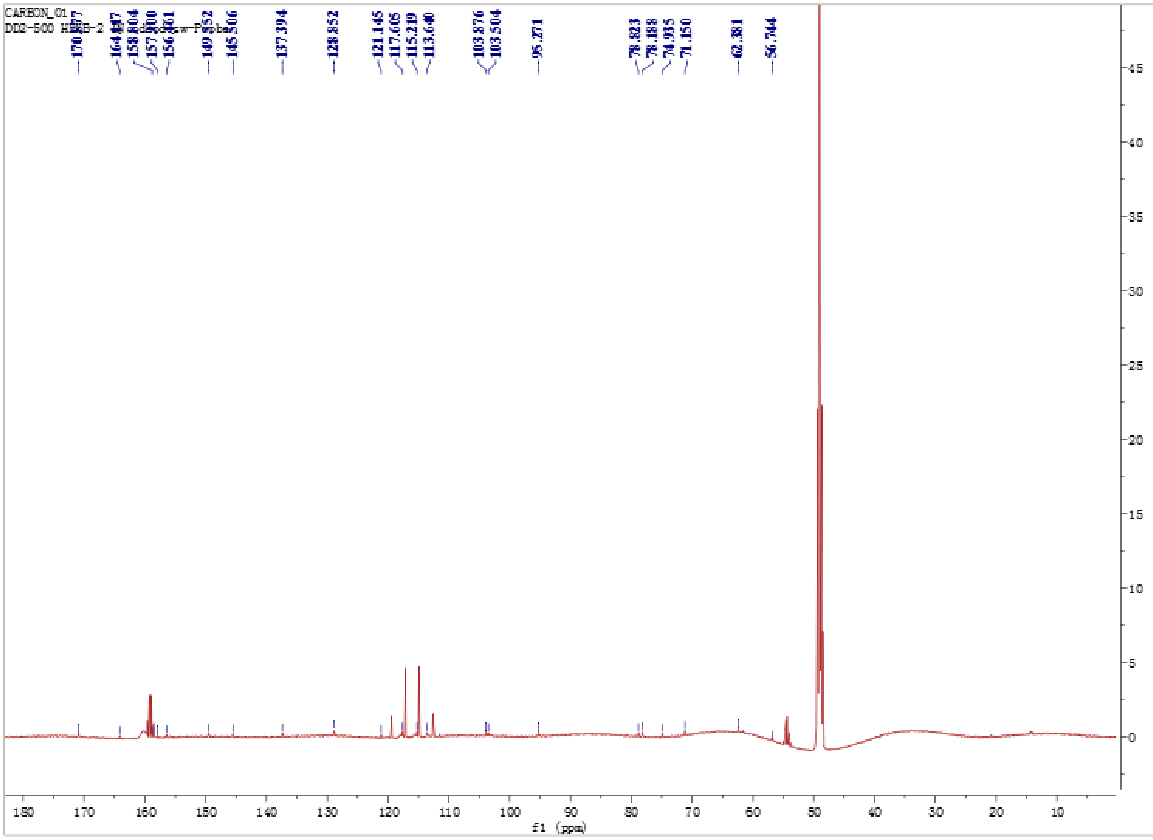

Figure S9. ^{1}H-NMR Spectrum (500 MHz, CD_3OD) of Compound **5**.

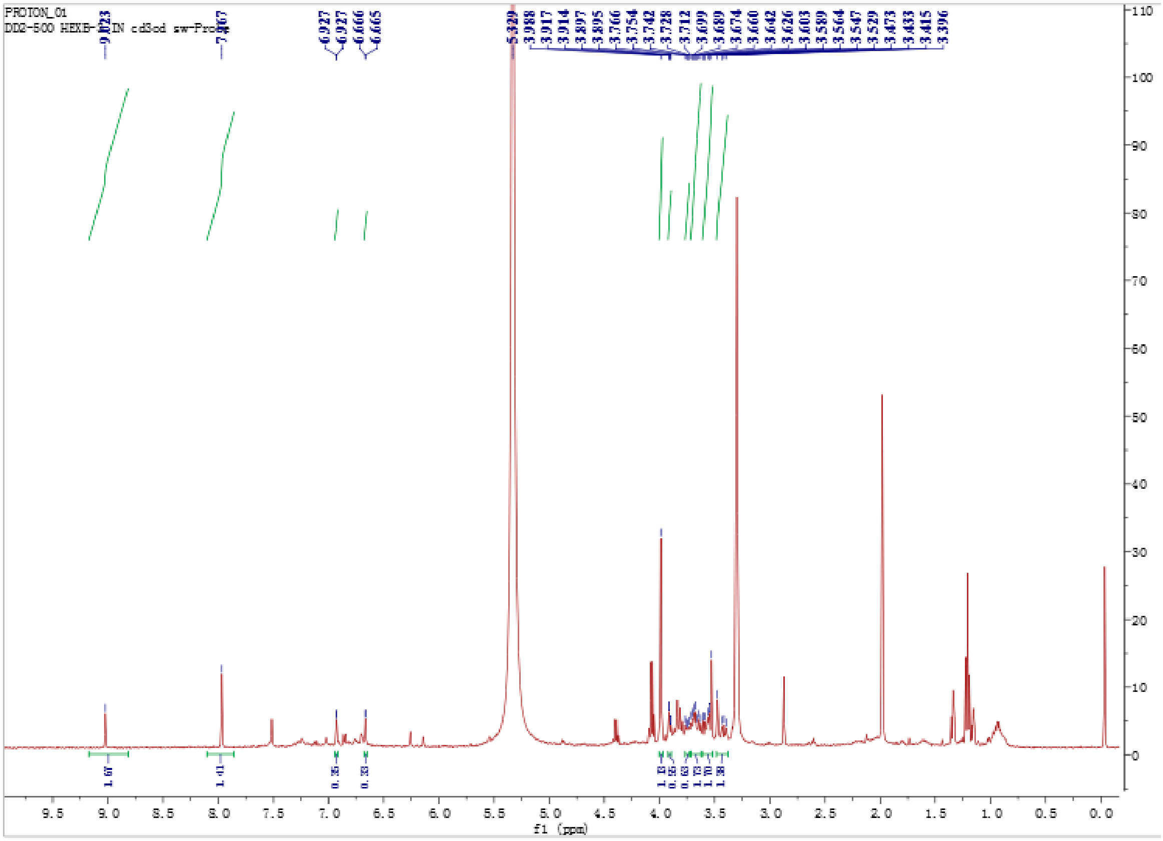

Figure S10. ^{13}C-NMR Spectrum (600 MHz, CD_3OD) of Compound **5**.

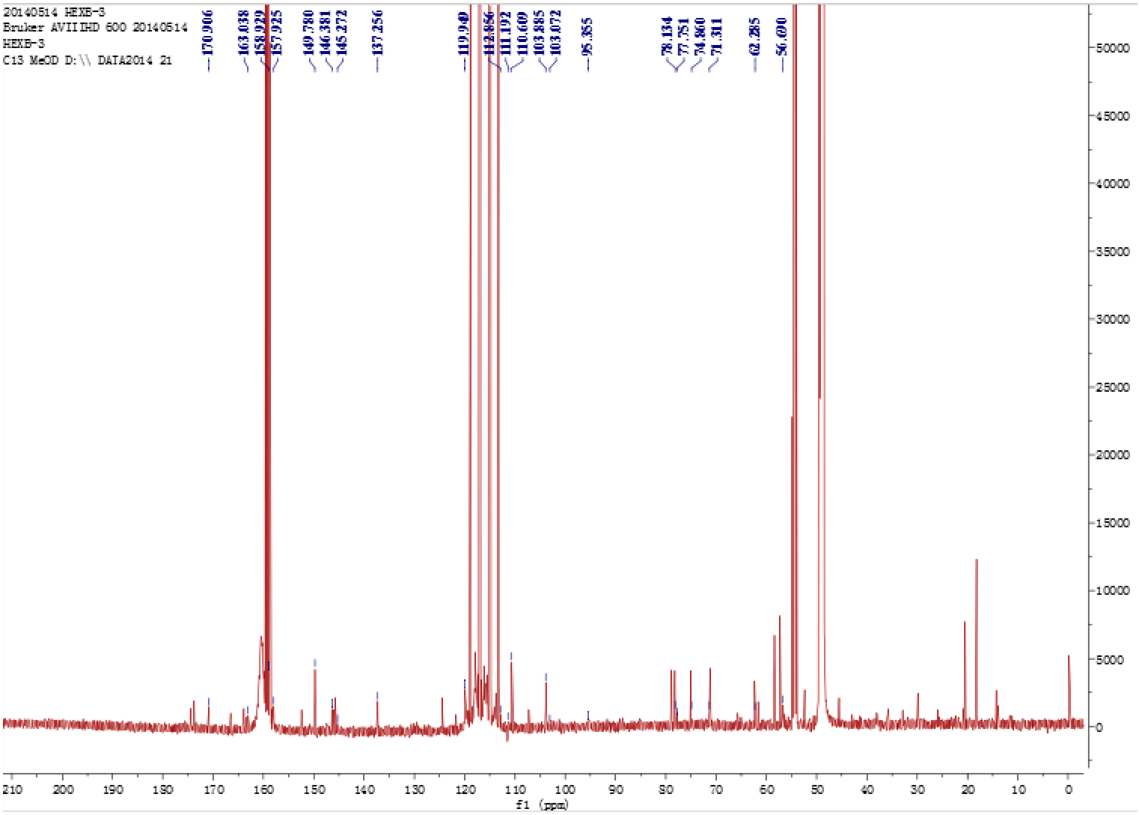

Qualitative Analysis Report

Data Filename	2014031203.d	Sample Name	HEXB-M-R-2
Sample Type	Sample	Position	P1-C1
Instrument Name	Instrument 1	User Name	
Acq Method		IRM Calibration Status	Success
DA Method	TEST LCMS.m	Comment	

User Chromatograms

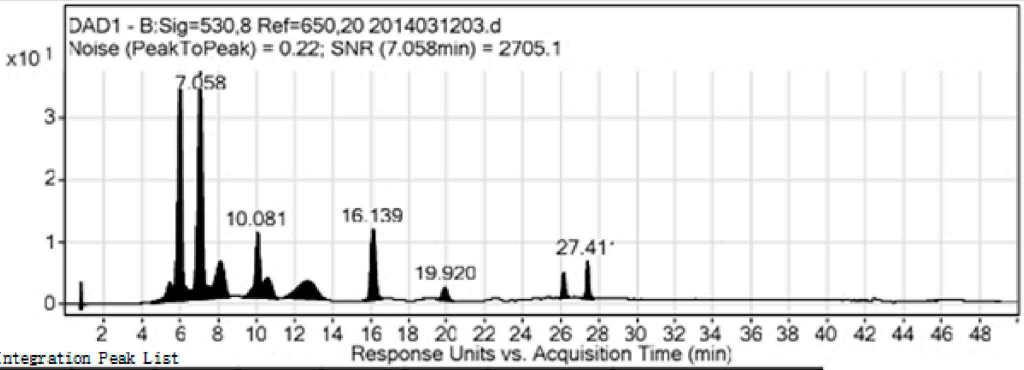

Integration Peak List

Peak	Start	RT	End	Height	Area	Area %	Signal To Noise
1	0.764	0.785	0.851	4.35	11.58	1.93	52.2
2	4.47	5.441	5.636	3.03	69.85	11.65	315
3	5.636	5.995	6.238	35.11	508.05	84.7	2291.3
6	6.588	7.058	7.365	36.65	599.8	100	2705.1
8	7.631	8.122	8.599	5.77	182.66	30.45	823.8
9	9.375	10.081	10.301	10.44	164.27	27.39	740.9
10	10.315	10.586	11.166	3.18	91.57	15.27	413
12	15.64	16.139	16.59	11.28	188.41	31.41	849.7
14	25.915	26.159	26.442	4.05	39.07	6.51	176.2
15	27.108	27.411	27.728	6.05	55.84	9.31	251.8

Noise Measurements

Noise Type	Signal Definition	Noise Multiplier	Noise Value
Peak-to-Peak	Area	1	0.221729279

Noise Regions

Start	End
2	2.5
17	17.3
21	21.5
35	36

Fragmentor Voltage 155 Collision Energy 0 Ionization Mode ESI

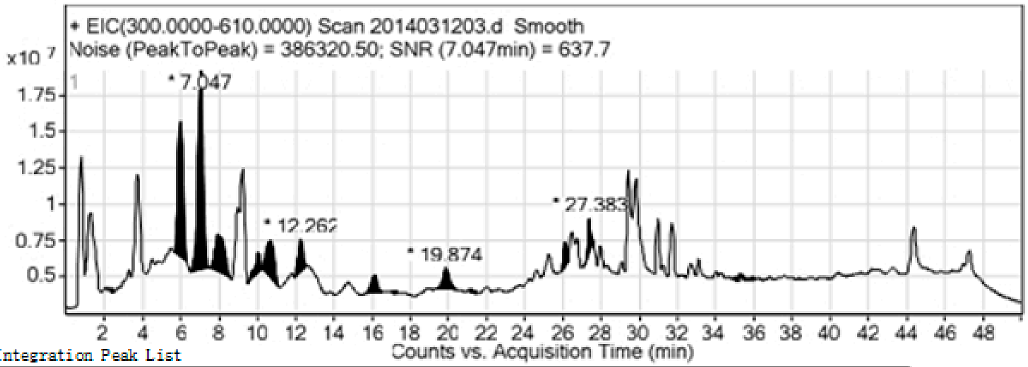

Integration Peak List

Peak	Start	RT	End	Height	Area	Area %	Signal To Noise

Qualitative Analysis Report

1	5.643	5.958	6.291	9305231	138761428	56.33	359.2
2	6.538	7.047	7.42	13649907	246355155	100	637.7
3	7.572	7.947	8.533	2550884	82236530	33.38	212.9
4	9.485	10.019	10.274	1388628	25939712	10.53	67.1
5	10.274	10.701	11.103	2522141	58645466	23.81	151.8
6	11.935	12.262	12.58	2184565	33458516	13.58	86.6
7	15.726	16.136	16.494	1195086	23994944	9.74	62.1
8	19.358	19.874	20.391	1440409	32571062	13.22	84.3
9	25.946	26.122	26.305	1671097	16852523	6.84	43.6
10	27.139	27.383	27.565	2216671	29388656	11.93	76.1

Noise Measurements

Noise Type	Signal Definition	Noise Multiplier	Noise Value
Peak-to-Peak	Area	1	386320.5

Noise Regions

Start	End
2	2.5
17	17.3
21	21.5
35	36

User Spectra

Fragmentor Voltage	Collision Energy	Ionization Mode
155	0	Esi

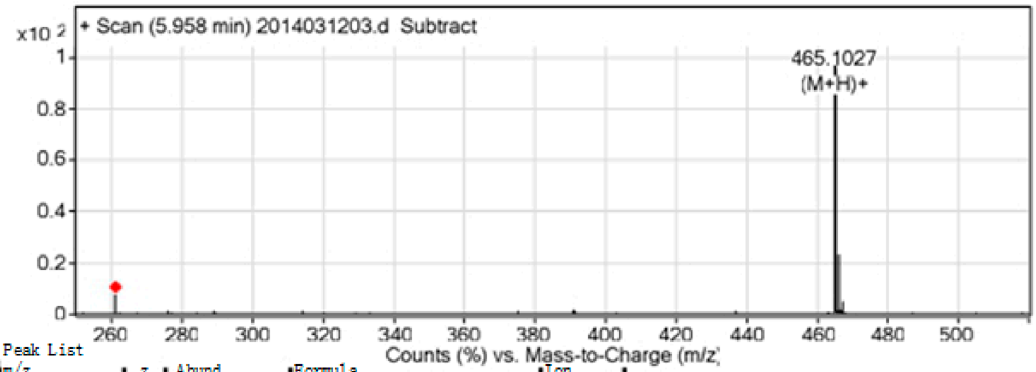

Peak List

m/z	z	Abund	Formula	Ion
103.0391		69327		
117.0547		68615		
177.0753		176012		
199.0574		446619		
261.0273		96862		
465.1027	1	1247393	C21 H21 O12	(M+H)+
465.283		76112		
466.1066	1	294210	C21 H21 O12	(M+H)+
467.1087	1	63460	C21 H21 O12	(M+H)+

Formula Calculator Element Limits

Element	Min	Max
C	3	100
H	0	500
O	0	90
N	0	5
S	0	0
Cl	0	0
Br	0	0
Si	0	0
F	0	0
Element	Min	Max

Qualitative Analysis Report

P	0	0

Formula Calculator Results

Formula	Best	Mass	Tgt Mass	Diff (ppm)	Ion Species	Score
C21 H20 O12	TRUE	464.0955	464.0955	0.01	C21 H21 O12	99.99
C19 H18 N3 O11		464.0955	464.0941	-2.89	C19 H19 N3 O11	99.8
C22 H16 N4 O8		464.0955	464.0968	2.88	C22 H17 N4 O8	99.7
C24 H18 N O9		464.0955	464.0982	5.78	C24 H19 N O9	99.17
C25 H20 O7 S		464.0955	464.093	-5.39	C25 H21 O7 S	98.48
C16 H22 N3 O11 S		464.0955	464.0975	4.37	C16 H23 N3 O11 S	98.43
C26 H16 N4 O3 S		464.0955	464.0943	-2.51	C26 H17 N4 O3 S	98.43
C28 H18 N O4 S		464.0955	464.0957	0.39	C28 H19 N O4 S	98.18
C20 H22 N3 O6 S2		464.0955	464.095	-1.03	C20 H23 N3 O6 S2	97.64
C22 H24 O7 S2		464.0955	464.0963	1.87	C22 H25 O7 S2	97.49

Fragmentor Voltage	Collision Energy	Ionization Mode
155	30	Esi

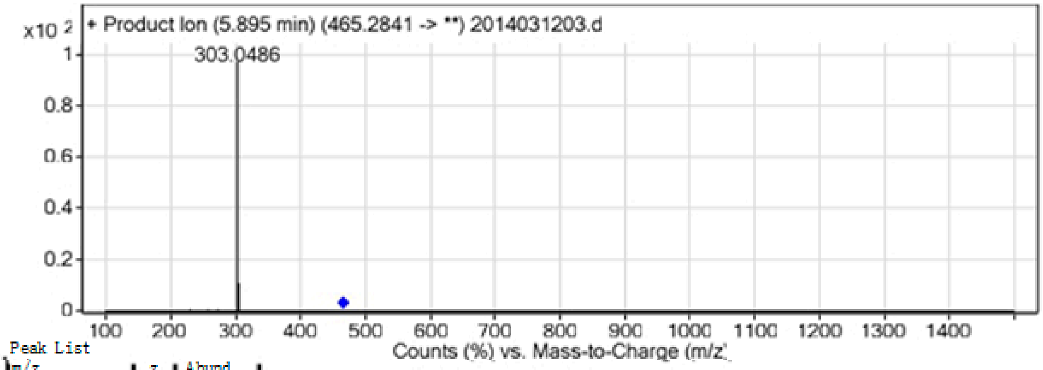

Peak List

m/z	z	Abund
303.0486	1	178656
304.0508	1	18712

Formula Calculator Element Limits

Element	Min	Max
C	3	100
H	0	500
O	0	90
N	0	5
S	0	0
Cl	0	0
Br	0	0
Si	0	0
F	0	0
P	0	0

Fragmentor Voltage	Collision Energy	Ionization Mode
155	0	Esi

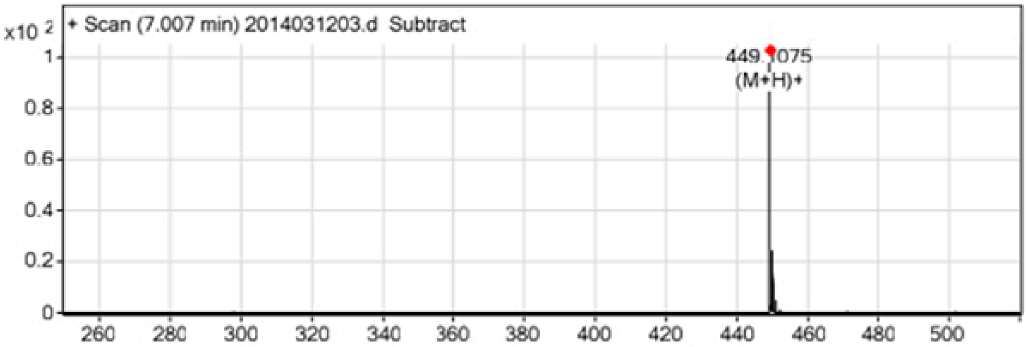

Qualitative Analysis Report

Peak List

m/z	z	Abund	Formula	Ion
449.1075	1	1648556	C21 H21 O11	(M+H)+
449.2871		109469		
450.1116	1	406326	C21 H21 O11	(M+H)+
451.1128	1	82613	C21 H21 O11	(M+H)+

Formula Calculator Element Limits

Element	Min	Max
C	3	100
H	0	500
O	0	90
N	0	5
S	0	0
Cl	0	0
Br	0	0
Si	0	0
F	0	0
P	0	0

Formula Calculator Results

Formula	Best	Mass	Tgt Mass	Diff (ppm)	Ion Species	Score
C21 H20 O11	TRUE	448.1003	448.1006	0.63	C21 H21 O11	99.94
C19 H18 N3 O10		448.1003	448.0992	-2.38	C19 H19 N3 O10	99.73
C22 H16 N4 O7		448.1003	448.1019	3.6	C22 H17 N4 O7	99.71
C26 H16 N4 O2 S		448.1003	448.0994	-1.98	C26 H17 N4 O2 S	98.76
C25 H20 O6 S		448.1003	448.0981	-4.96	C25 H21 O6 S	98.74
C28 H18 N O3 S		448.1003	448.1007	1.02	C28 H19 N O3 S	98.5
C16 H22 N3 O10 S		448.1003	448.1026	5.14	C16 H23 N3 O10 S	98.04
C20 H22 N3 O5 S2		448.1003	448.1001	-0.45	C20 H23 N3 O5 S2	97.62
C22 H24 O6 S2		448.1003	448.1014	2.56	C22 H25 O6 S2	97.46
C23 H20 N4 O2 S2		448.1003	448.1028	5.53	C23 H21 N4 O2 S2	97.25

Fragmentor Voltage	Collision Energy	Ionization Mode
155	30	Esi

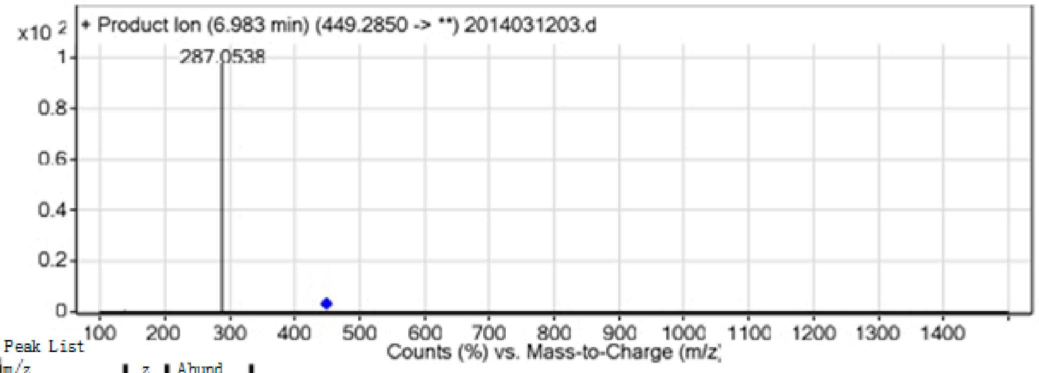

Peak List

m/z	z	Abund
287.0538	1	267384
287.1966		13466
288.0569	1	29125

Formula Calculator Element Limits

Element	Min	Max
C	3	100
H	0	500
O	0	90
N	0	5
S	0	0
Cl	0	0
Br	0	0

Qualitative Analysis Report

Element	Min	Max
Si	0	0
F	0	0
P	0	0

Fragmentor Voltage	Collision Energy	Ionization Mode
155	0	Esi

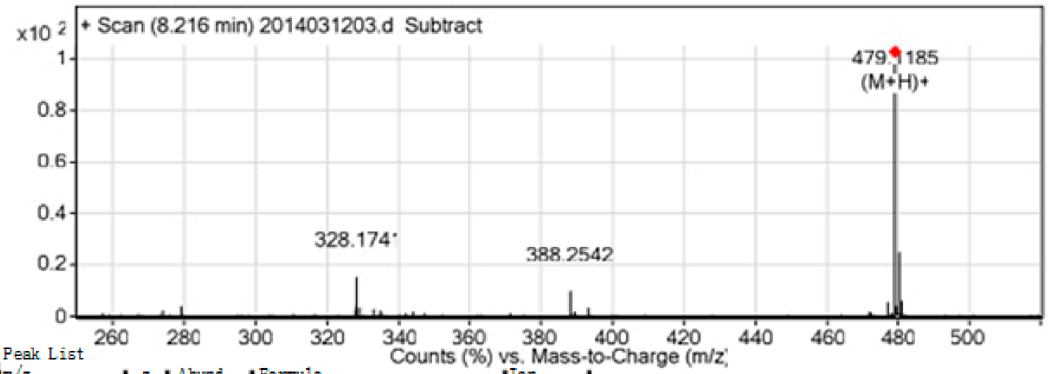

Peak List

m/z	z	Abund	Formula	Ion
328.1741		34593		
388.2542		22923		
477.1187		13271		
479.1185	1	230115	C22 H23 O12	(M+H)+
480.1215	1	57123	C22 H23 O12	(M+H)+
481.1229	1	13627	C22 H23 O12	(M+H)+

Formula Calculator Element Limits

Element	Min	Max
C	3	100
H	0	500
O	0	90
N	0	5
S	0	0
Cl	0	0
Br	0	0
Si	0	0
F	0	0
P	0	0

Formula Calculator Results

Formula	Best	Mass	Tgt Mass	Diff (ppm)	Ion Species	Score
C22 H22 O12	TRUE	478.1113	478.1111	-0.27	C22 H23 O12	99.97
C20 H20 N3 O11		478.1113	478.1098	-3.08	C20 H21 N3 O11	99.77
C23 H18 N4 O8		478.1113	478.1125	2.52	C23 H19 N4 O8	99.73
C25 H20 N O9		478.1113	478.1138	5.34	C25 H21 N O9	99.25
C17 H24 N3 O11 S		478.1113	478.1132	3.96	C17 H25 N3 O11 S	98.71
C26 H22 O7 S		478.1113	478.1086	-5.51	C26 H23 O7 S	98.69
C27 H18 N4 O3 S		478.1113	478.11	-2.72	C27 H19 N4 O3 S	98.66
C29 H20 N O4 S		478.1113	478.1113	0.1	C29 H21 N O4 S	98.45
C21 H24 N3 O6 S2		478.1113	478.1107	-1.28	C21 H25 N3 O6 S2	98.02
C23 H26 O7 S2		478.1113	478.112	1.54	C23 H27 O7 S2	97.88

Fragmentor Voltage	Collision Energy	Ionization Mode
155	30	Esi

Qualitative Analysis Report

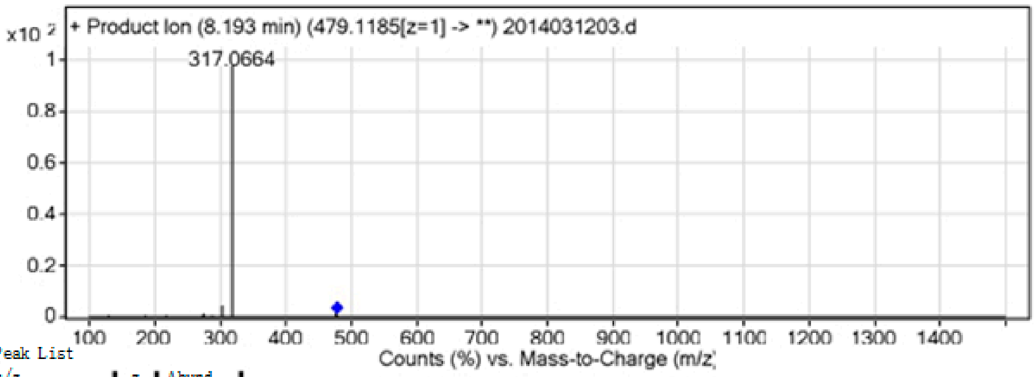

Peak List

m/z	z	Abund
317.0664	1	41425
318.0695	1	4558

Formula Calculator Element Limits

Element	Min	Max
C	3	100
H	0	500
O	0	90
N	0	5
S	0	0
Cl	0	0
Br	0	0
Si	0	0
F	0	0
P	0	0

Fragmentor Voltage	Collision Energy	Ionization Mode
155	0	Esi

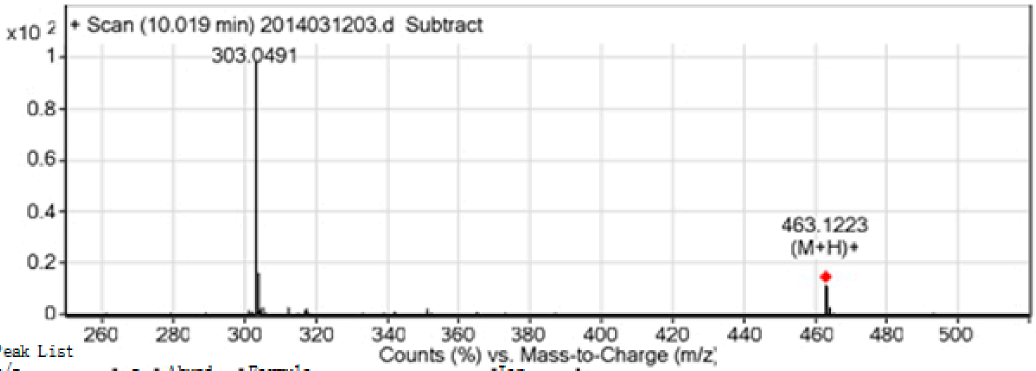

Peak List

m/z	z	Abund	Formula	Ion
185.0426		20088		
303.0491	1	267569		
304.0526	1	45089		
463.1223		30856	C22 H23 O11	(M+H)+
565.1183		27505		

Formula Calculator Element Limits

Element	Min	Max
C	3	100
H	0	500
O	0	90
N	0	5
S	0	0
Cl	0	0
Br	0	0

Qualitative Analysis Report

Element	Min	Max
Si	0	0
F	0	0
P	0	0

Formula Calculator Results

Formula	Best	Mass	Tgt Mass	Diff (ppm)	Ion Species	Score
C20 H20 N3 O10		462.115	462.1149	-0.25	C20 H21 N3 O10	99.86
C22 H22 O11	TRUE	462.115	462.1162	2.67	C22 H23 O11	99.64
C23 H18 N4 O7		462.115	462.1175	5.55	C23 H19 N4 O7	98.85
C32 H16 N O3		462.115	462.113	-4.25	C32 H17 N O3	96.04
C35 H14 N2		462.115	462.1157	1.55	C35 H15 N2	94.63

Fragmentor Voltage	Collision Energy	Ionization Mode
155	30	Esi

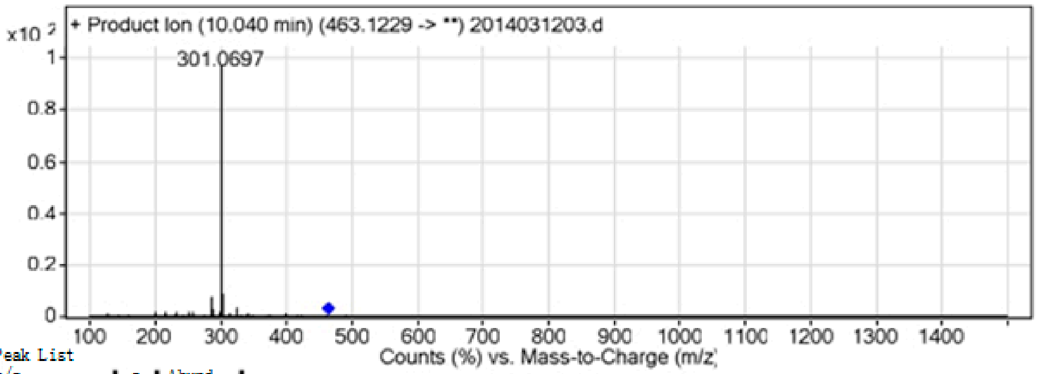

Peak List

m/z	z	Abund
286.0464		401
301.0697	1	5153
302.0763	1	448

Formula Calculator Element Limits

Element	Min	Max
C	3	100
H	0	500
O	0	90
N	0	5
S	0	0
Cl	0	0
Br	0	0
Si	0	0
F	0	0
P	0	0

Fragmentor Voltage	Collision Energy	Ionization Mode
155	0	Esi

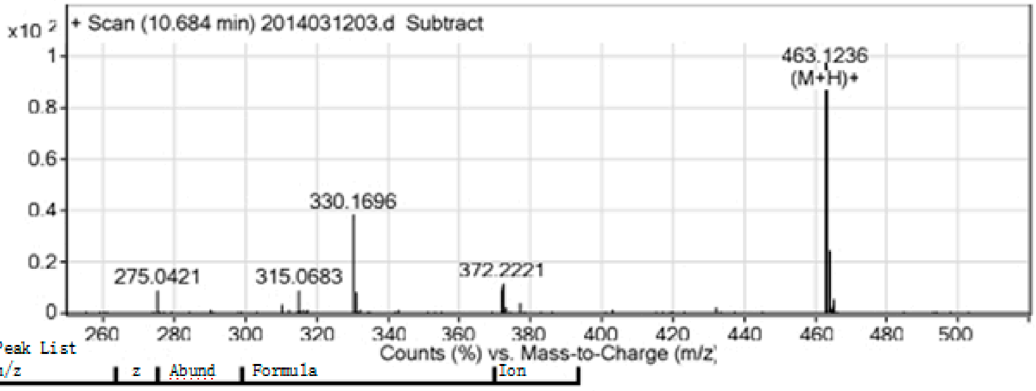

Peak List

m/z	z	Abund	Formula	Ion

Qualitative Analysis Report

117.0546		32605		
191.0911		47238		
213.0726		109922		
275.0421		19304		
315.0683		19582		
330.1696	1	86073		
331.1726	1	18597		
372.2221		25795		
463.1236	1	224838	C22 H23 O11	(M+H)+
464.1268	1	55610	C22 H23 O11	(M+H)+

Formula Calculator Element Limits

Element	Min	Max
C	3	100
H	0	500
O	0	90
N	0	5
S	0	0
Cl	0	0
Br	0	0
Si	0	0
F	0	0
P	0	0

Formula Calculator Results

Formula	Best	Mass	Tgt Mass	Diff (ppm)	Ion Species	Score
C22 H22 O11	TRUE	462.1163	462.1162	-0.28	C22 H23 O11	99.96
C20 H20 N3 O10		462.1163	462.1149	-3.19	C20 H21 N3 O10	99.74
C23 H18 N4 O7		462.1163	462.1175	2.61	C23 H19 N4 O7	99.74
C25 H20 N O8		462.1163	462.1189	5.52	C25 H21 N O8	99.24
C35 H14 N2		462.1163	462.1157	-1.39	C35 H15 N2	96.27

Fragmentor Voltage	Collision Energy	Ionization Mode
155	30	Esi

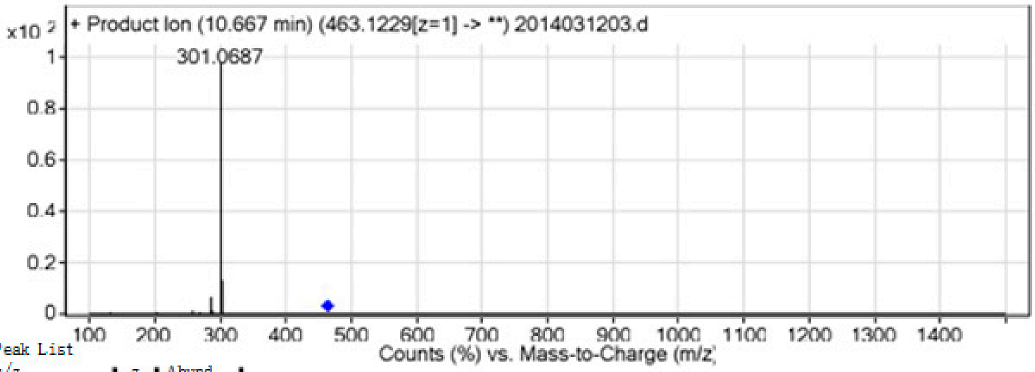

Peak List

m/z	z	Abund
286.0452		2542
301.0687	1	38360
302.0737	1	4892

Formula Calculator Element Limits

Element	Min	Max
C	3	100
H	0	500
O	0	90
N	0	5
S	0	0
Cl	0	0
Element	Min	Max
Br	0	0

Qualitative Analysis Report

Si	0	0
F	0	0
P	0	0

Fragmentor Voltage	Collision Energy	Ionization Mode
155	0	Esi

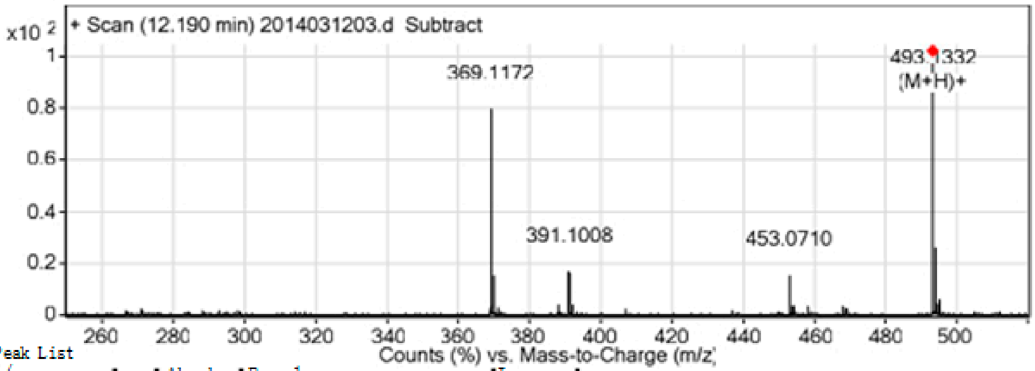

Peak List

m/z	z	Abund	Formula	Ion
163.0386		18728		
204.0372		10805		
224.5494	2	54125		
225.0505	2	14119		
369.1172	1	98097		
370.1205	1	18943		
391.1008		20630		
453.071		18537		
493.1332	1	121765	C23 H25 O12	(M+H)+
494.1369	1	32098	C23 H25 O12	(M+H)+

Formula Calculator Element Limits

Element	Min	Max
C	3	100
H	0	500
O	0	90
N	0	5
S	0	0
Cl	0	0
Br	0	0
Si	0	0
F	0	0
P	0	0

Formula Calculator Results

Formula	Best	Mass	Tgt Mass	Diff (ppm)	Ion Species	Score
C23 H24 O12	TRUE	492.126	492.1268	1.64	C23 H25 O12	99.94
C21 H22 N3 O11		492.126	492.1254	-1.1	C21 H23 N3 O11	99.88
C24 H20 N4 O8		492.126	492.1281	4.35	C24 H21 N4 O8	99.59
C33 H18 N O4		492.126	492.1236	-4.85	C33 H19 N O4	97.6
C34 H14 N5		492.126	492.1249	-2.14	C34 H15 N5	96.96
C36 H16 N2 O		492.126	492.1263	0.59	C36 H17 N2 O	96.64

Fragmentor Voltage	Collision Energy	Ionization Mode
155	30	Esi

Qualitative Analysis Report

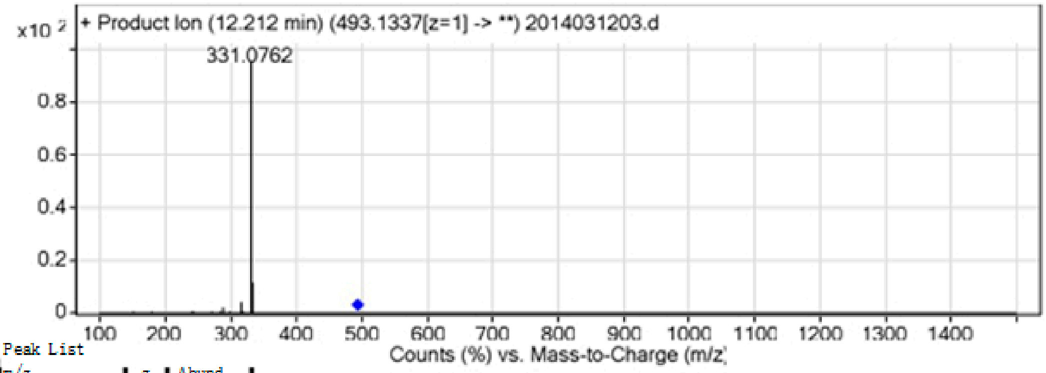

Peak List

m/z	z	Abund
331.0762	1	26264
332.0805	1	3054

Formula Calculator Element Limits

Element	Min	Max
C	3	100
H	0	500
O	0	90
N	0	5
S	0	0
Cl	0	0
Br	0	0
Si	0	0
F	0	0
P	0	0

Fragmentor Voltage	Collision Energy	Ionization Mode
155	0	Esi

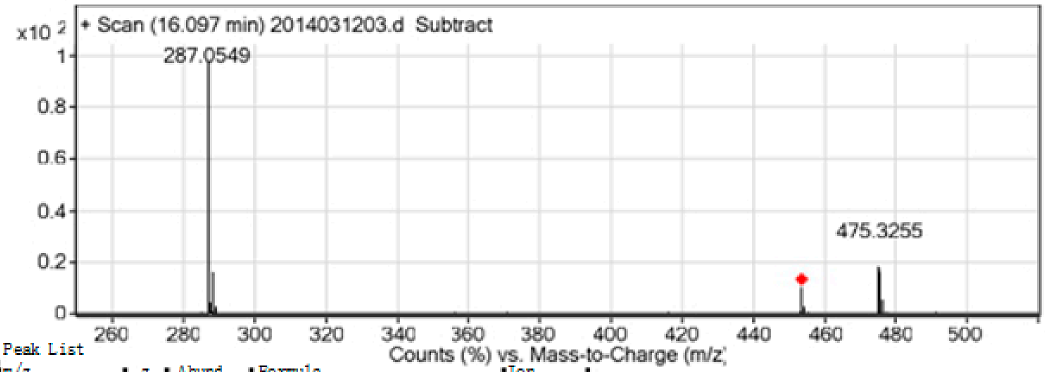

Peak List

m/z	z	Abund	Formula	Ion
287.0549	1	514308		
288.0585	1	85097		
453.3435		52063	C23 H49 O8	(M+H)+
475.3255	1	95391		
476.3281	1	28057		

Formula Calculator Element Limits

Element	Min	Max
C	3	100
H	0	500
O	0	90
N	0	5
S	0	0
Cl	0	0
Br	0	0

Qualitative Analysis Report

Element	Min	Max
Si	0	0
F	0	0
P	0	0

Formula Calculator Results

Formula	Best	Mass	Tgt Mass	Diff (ppm)	Ion Species	Score
C24 H44 N4 O4		452.3363	452.3363	0	C24 H45 N4 O4	99.97
C26 H46 N O5		452.3363	452.3376	2.98	C26 H47 N O5	99.77
C23 H48 O8	TRUE	452.3362	452.3349	-2.94	C23 H49 O8	99.72
C21 H46 N3 O7		452.3363	452.3336	-5.92	C21 H47 N3 O7	99.18
C27 H42 N5 O		452.3363	452.3389	5.92	C27 H43 N5 O	99.07

Fragmentor Voltage	Collision Energy	Ionization Mode
155	30	Esi

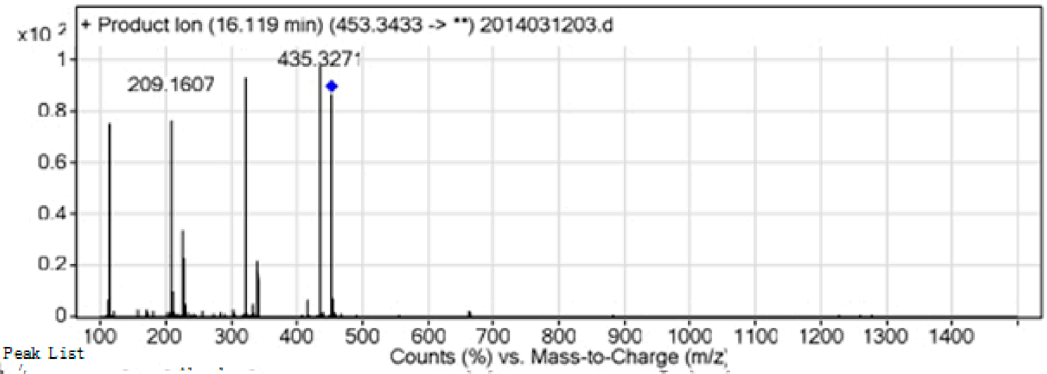

Peak List

m/z	z	Abund
114.0897	1	1483
209.1607	1	1523
226.1892		658
227.1726		274
228.1572		448
322.2457		1820
339.2755		419
340.2602		396
435.3271	1	1952
453.336	1	1691

Formula Calculator Element Limits

Element	Min	Max
C	3	100
H	0	500
O	0	90
N	0	5
S	0	0
Cl	0	0
Br	0	0
Si	0	0
F	0	0
P	0	0

Fragmentor Voltage	Collision Energy	Ionization Mode
155	0	Esi

Qualitative Analysis Report

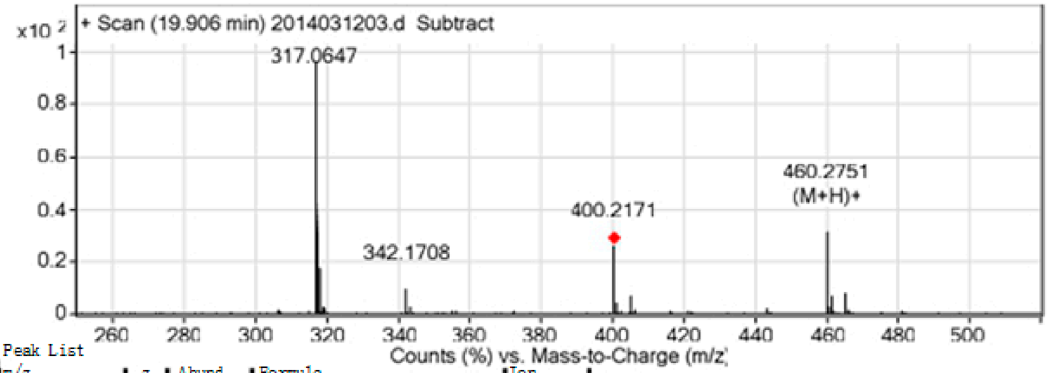

Peak List

m/z	z	Abund	Formula	Ion
121.0455		18530		
317.0647	1	119037		
318.0686	1	20786		
342.1708		11609		
400.2171		31282		
405.1738		8450		
460.2751	1	37780	C19 H42 N O11	(M+H)+
461.2791	1	8425	C19 H42 N O11	(M+H)+
465.2301		9919		

Formula Calculator Element Limits

Element	Min	Max
C	3	100
H	0	500
O	0	90
N	0	5
S	0	0
Cl	0	0
Br	0	0
Si	0	0
F	0	0
P	0	0

Formula Calculator Results

Formula	Best	Mass	Tgt Mass	Diff (ppm)	Ion Species	Score
C19 H41 N O11	TRUE	459.2678	459.268	0.37	C19 H42 N O11	99.95
C17 H39 N4 O10		459.2678	459.2666	-2.56	C17 H40 N4 O10	99.77
C20 H37 N5 O7		459.2678	459.2693	3.27	C20 H38 N5 O7	99.67
C32 H33 N3		459.2678	459.2674	-0.75	C32 H34 N3	95.91
C34 H35 O		459.2678	459.2688	2.18	C34 H36 O	95.37

Fragmentor Voltage	Collision Energy	Ionization Mode
155	30	Esi

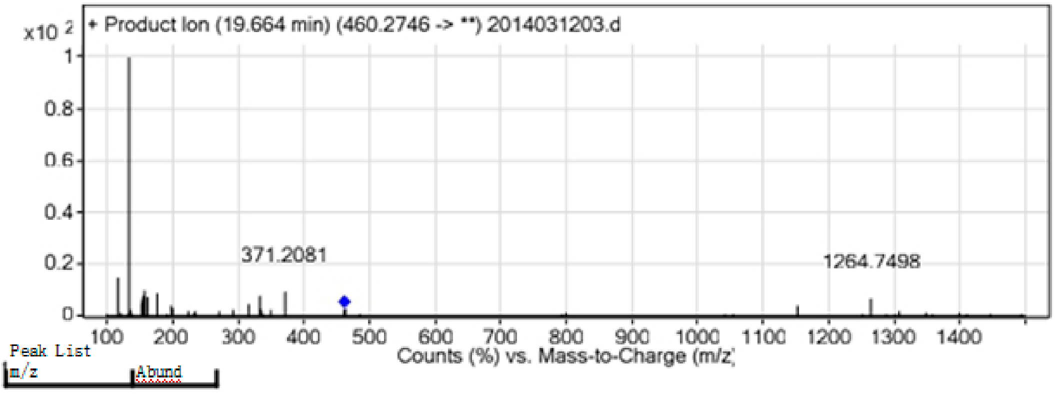

Peak List

m/z	Abund

Qualitative Analysis Report

117.0883	96
133.087	654
153.9169	50
156.8868	64
161.0778	48
177.1082	58
333.8117	48
371.2081	62
1264.7498	43

Formula Calculator Element Limits

Element	Min	Max
C	3	100
H	0	500
O	0	90
N	0	5
S	0	0
Cl	0	0
Br	0	0
Si	0	0
F	0	0
P	0	0

Fragmentor Voltage	Collision Energy	Ionization Mode
155	0	Esi

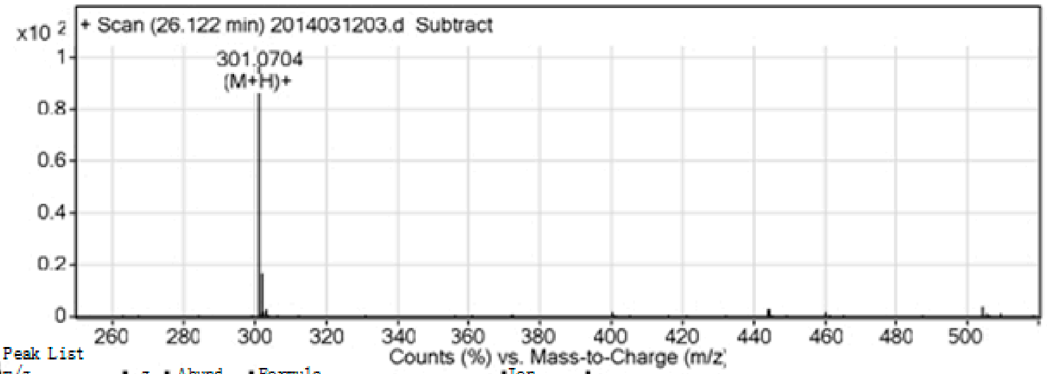

Peak List

m/z	z	Abund	Formula	Ion
301.0704	1	405070	C16 H13 O6	(M+H)+
302.0737	1	69280	C16 H13 O6	(M+H)+

Formula Calculator Element Limits

Element	Min	Max
C	3	100
H	0	500
O	0	90
N	0	5
S	0	0
Cl	0	0
Br	0	0
Si	0	0
F	0	0
P	0	0

Formula Calculator Results

Formula	Best	Mass	Tgt Mass	Diff (ppm)	Ion Species	Score
C16 H12 O6	TRUE	300.0632	300.0634	0.77	C16 H13 O6	99.97
C14 H10 N3 O5		300.0632	300.062	-3.72	C14 H11 N3 O5	99.77
C17 H8 N4 O2		300.0632	300.0647	5.21	C17 H9 N4 O2	99.29

Fragmentor Voltage	Collision Energy	Ionization Mode
155	30	Esi

Qualitative Analysis Report

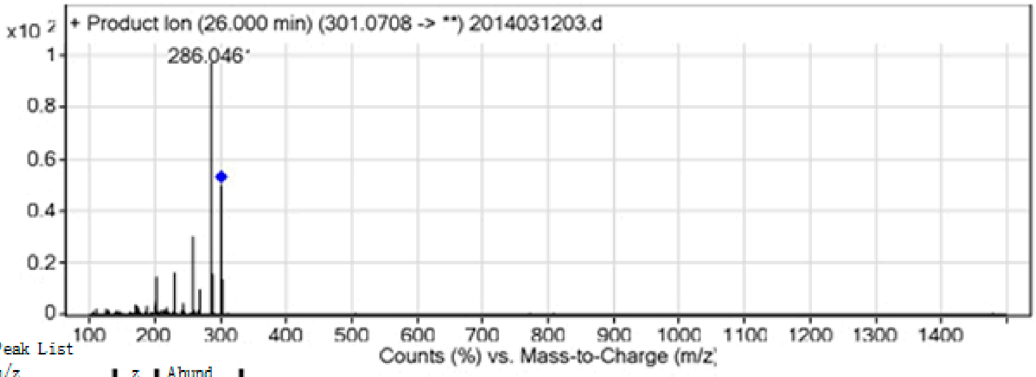

Peak List

m/z	z	Abund
202.0596		718
229.045		648
230.0538		810
257.0402		736
258.0497		1476
268.0354		473
286.0461	1	4900
287.0444	1	760
301.0678	1	2484
302.0696	1	666

Formula Calculator Element Limits

Element	Min	Max
C	3	100
H	0	500
O	0	90
N	0	5
S	0	0
Cl	0	0
Br	0	0
Si	0	0
F	0	0
P	0	0

Fragmentor Voltage	Collision Energy	Ionization Mode
155	0	Esi

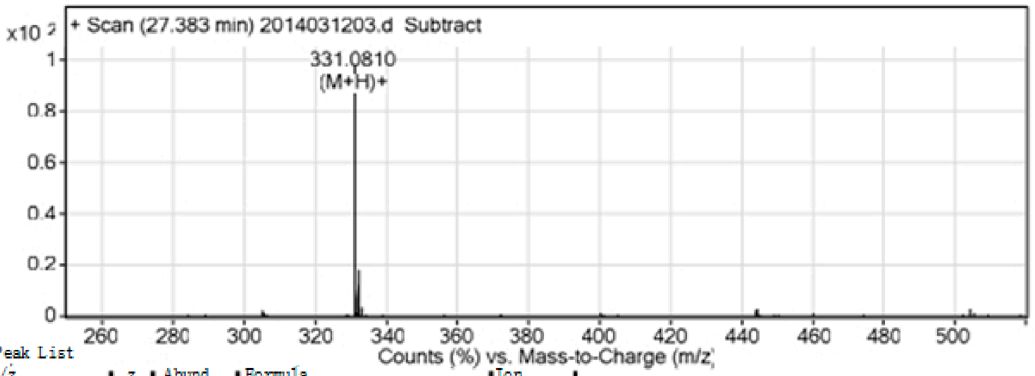

Peak List

m/z	z	Abund	Formula	Ion
331.081	1	491513	C17 H15 O7	(M+H)+
332.0839	1	88997	C17 H15 O7	(M+H)+

Formula Calculator Element Limits

Element	Min	Max
C	3	100
H	0	500

Qualitative Analysis Report

O	0	90
N	0	5
S	0	0
Cl	0	0
Br	0	0
Si	0	0
F	0	0
P	0	0

Formula Calculator Results

Formula	Best	Mass	Tgt Mass	Diff (ppm)	Ion Species	Score
C17 H14 O7	TRUE	330.0737	330.074	0.63	C17 H15 O7	99.96
C15 H12 N3 O6		330.0737	330.0726	-3.45	C15 H13 N3 O6	99.8
C18 H10 N4 O3		330.0737	330.0753	4.67	C18 H11 N4 O3	99.35

Fragmentor Voltage	Collision Energy	Ionization Mode
155	30	Esi

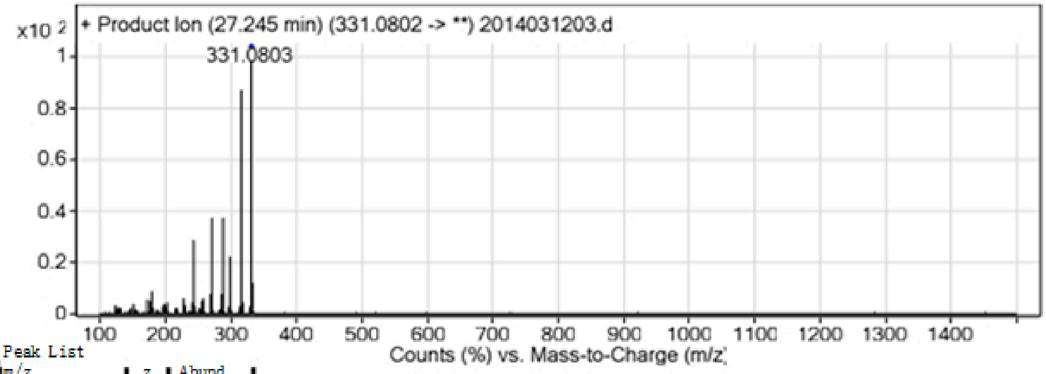

Peak List

m/z	z	Abund
178.022		228
242.0547		732
270.0525		954
287.0557		971
298.0431		572
299.0503		433
315.0479	1	2268
316.051	1	586
331.0803	1	2550
332.0863	1	304

Formula Calculator Element Limits

Element	Min	Max
C	3	100
H	0	500
O	0	90
N	0	5
S	0	0
Cl	0	0
Br	0	0
Si	0	0
F	0	0
P	0	0

--- End Of Report ---
